# Construction of High‐Performance Anode of Potassium‐Ion Batteries by Stripping Covalent Triazine Frameworks with Molten Salt

**DOI:** 10.1002/advs.202401804

**Published:** 2024-06-26

**Authors:** Jingyi Zhang, Xuwang Fu, Jiacheng Qiu, Chao Wang, Li Wang, Jianmin Feng, Lei Dong, Conglai Long, Xiaowei Wang, Dejun Li

**Affiliations:** ^1^ College of Physics and Materials Science Tianjin Normal University Tianjin 300387 China; ^2^ National Engineering Laboratory for High Efficiency Recovery of Refractory Nonferrous Metals School of Metallurgy and Environment Central South University Changsha 410083 P. R. China

**Keywords:** covalent triazine frameworks, melt‐salt‐stripping, potassium‐ion batteries

## Abstract

Covalent triazine frameworks (CTFs) are promising battery electrodes owing to their designable functional groups, tunable pore sizes, and exceptional stability. However, their practical use is limited because of the difficulty in establishing stable ion adsorption/desorption sites. In this study, a melt‐salt‐stripping process utilizing molten trichloro iron (FeCl_3_) is used to delaminate the layer‐stacked structure of fluorinated covalent triazine framework (FCTF) and generate iron‐based ion storage active sites. This process increases the interlayer spacing and uniformly deposits iron‐containing materials, enhancing electron and ion transport. The resultant melt‐FeCl_3_‐stripped FCTF (Fe@FCTF) shows excellent performance as a potassium ion battery with a high capacity of 447 mAh g^−1^ at 0.1 A g^−1^ and 257 mAh g^−1^ at 1.6 A g^−1^ and good cycling stability. Notably, molten‐salt stripping is also effective in improving the CTF's Na^+^ and Li^+^ storage properties. A stepwise reaction mechanism of K/Na/Li chelation with C═N functional groups is proposed and verified by in situ X‐ray diffraction testing (XRD), ex‐situ X‐ray photoelectron spectroscopy (XPS), and theoretical calculations, illustrating that pyrazines and iron coordination groups play the main roles in reacting with K^+^/Na^+^/Li^+^ cations. These results conclude that the Fe@FCTF is a suitable anode material for potassium‐ion batteries (PIBs), sodium‐ion batteries (SIBs), and lithium‐ion batteries (LIBs).

## Graphical abstract

A novel covalent organic framework, melt‐FeCl_3_‐stripped FCTF (Fe@FCTF), is designed via a salt‐stripping process using molten ferric chloride (FeCl_3_). The stripped samples resembled petal shapes, opening ion channels and decreasing the energy band gap, demonstrating excellent ionic energy storage capacity and good cycling stability (85% specific capacitance retention after 500 cycles).

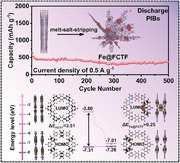

## 1 Introduction

Driven by the energy‐storage industry, the development of new energy‐storage systems is urgently required. LIBs have a high energy density, excellent cycle stability, and extremely low self‐discharge rates, making them one of the best energy storage solutions.^[^ ^1^, ^2^, ^3^ ^]^ However, due to lithium resources scarcity, LIBs have difficulty meeting the huge demand for new energy‐storage devices.^[^ ^4^, ^5^, ^6^ ^]^ As a result, the development of secondary PIBs has gained wide attention, as they replace Li^+^ with K^+,^ which is abundant and easily accessible.^[^ ^7^, ^8^, ^9^ ^]^ Although the energy storage mechanism of PIBs is the same as that of LIBs, the larger size of K^+^ makes it difficult for K^+^ to embed and migrate in the electrode, limiting the electrode capacity improvement.^[^ ^10^, ^11^, ^12^, ^13^ ^]^ Moreover, the insertion/extraction of K^+^ causes significant volume changes in the electrode, causing the active material to be pulverized and detached from the collector, resulting in battery failure.^[^ ^14^, ^15^, ^16^ ^]^ Therefore, the key to the widespread application of PIBs is the development of high‐performance electrode materials suitable for K^+^ storage. Anode materials such as carbon‐based, oxide, and alloy materials have been extensively explored as an essential part of PIBs.^[^ ^17^, ^18^, ^19^ ^]^ Carbon‐based materials have the advantages of good structural stability, good electrical conductivity, low cost, and low environmental pollution. However, when used to store large amounts of K^+^, traditional carbon‐based materials have low capacity and large volume change, leading to low cycle stability.

Currently, the main strategies to solve this problem are to construct nano‐structures suitable for K^+^ migration and enrich the K^+^ storage active sites by doping.^[^ ^20^, ^21^ ^]^ Nano‐structure engineering and doping processes often involve complicated steps, such as precursor design, template introduction, repeated sintering, and template removal, which not only increase the manufacturing cost of the material but also pose significant challenges to its controllability.^[^ ^22^, ^23^ ^]^ In particular, during sintering, the structure of the material changes dramatically, making it difficult to adjust the nanostructure, and inevitable ion diffusion dead zones limit the increase in material's K^+^ storage capacity.^[^ ^24^, ^25^ ^]^

Covalent triazine frameworks (CTFs) based on carbon‐skeleton structures have shown great potential for Li storage because of their multifunctional characteristics. CTFs have rich and adjustable pore structures (nitrogen (N═N), amides (C═N), carbonyl (C═O), and aromatic rings) for ion insertion/extraction and a large specific surface area.^[^ ^26^, ^27^, ^28^, ^29^, ^30^, ^31^, ^32^ ^]^ However, the insertion/extraction of K^+^ from CTFs remains a challenge, resulting in low K^+^ storage capacity, which limits the application of CTFs in PIBs.^[^ ^33^, ^34^, ^35^, ^36^, ^37^, ^38^ ^]^ To overcome this limitation, the CTF must be stripped, and an inorganic component is introduced to expand the channels that embed the ions and expose the active site.^[^ ^39^, ^40^, ^41^, ^42^ ^]^ Guo et al. employed a lithiation‐assisted chemical exfoliation technique to implement FL‐GP, effectively expanding the layer spacing after preliminary lithium‐ion intercalation. Fl‐GP/rGO demonstrated excellent sodium storage performance. With a 2D structure, FL‐GP shortens the ion transport path and mitigates volume change during the sodiation process.^[^ ^41^ ^]^ Liu et al. prepared recombinant MoS_2_ extending along the c‐axis via exfoliation and recombination.^[^ ^31^, ^43^, ^44^, ^45^, ^46^ ^]^ Guo et al. observed that pre‐intercalation is a versatile strategy that simultaneously stabilizes the structure and enhances the kinetics of cathodes. This method can incorporate various guests, such as metal ions and neutral molecules, to modulate the structural properties of cathode materials. The effectiveness of these strategies suggests significant potential to enhance cathode performance.^[^ ^3^, ^40^, ^47^ ^]^ Zhao et al. designed several layers of a COF material (COF‐Co) as an anode for PIBs based on the binding properties of cyanide groups with cobalt. The introduction of cobalt produced numerous defects, altering the π‐electronic structure of the benzene ring and enhancing the π‐K^+^ effect. As a result, COF‐Co showed an improved cycling capacity owing to the utilization of the interlayer active sites after stripping.^[^ ^27^ ^]^ These stripping‐related studies provide a viable strategy for efficiently utilizing COF active sites and promoting their energy storage application.^[^ ^48^, ^49^, ^50^, ^51^, ^52^, ^53^ ^]^ So far, only ball‐milling and chemical stripping have been reported, but they are still not sufficiently efficient and mild. Therefore, developing a mild and efficient CTF stripping process is essential for developing CTFs as PIBs electrode materials and for other functional applications.

Herein, we present a facile melt‐salt stripping method for stripping FCTFs using FeCl_3_. This process effectively delaminated the tightly stacked CTFs into a warp‐layered stacked structure, which facilitated the penetration of K^+^ into the redox sites and enhanced FCTF K^+^ storage performance. During this reaction, a portion of the iron‐containing species was successfully bonded to the surface of CTFs, providing additional reaction sites. However, the obtained FCTF‐Fe structure has rich defects originating from the introduction of coordinated Fe centers, which can improve the electronic conductivity and provide electronic conductivity to more K^+^ transfer channels. The resultant Fe@FCTF exhibited excellent K^+^ storage performance. When used as an anode for PIBs, it exhibited a high specific capacity of 447 mAh g^−1^ at 0.1 A g^−1^, an excellent rate performance of 203 mAh g^−1^ at 2 A g^−1^, and good cycling stability with a capacity retention rate close to 85% after 500 cycles. Notably, fluorine in the FCTF was beneficial for loading iron‐based K^+^ storage active sites. Compared to the non‐fluorine CTF, introducing F facilitated the uniform anchoring of Fe on the carbon skeleton, shortened the electron/ion diffusion paths, and improved the overall conductivity. Furthermore, the Fe@FCTF exhibited excellent storage performance of Na^+^ and Li^+^. The Na^+^ storage capacities of melt‐salt‐stipping CTF are kept at 438 mAh g^−1^ after 100 cycles at 0.1 A g^−1^. The Li^+^ storage capacities are kept at 1526 mAh g^−1^ after 100 cycles at 0.1 A g^−1^. The development of melt‐salt stipping promotes the battery application of CTFs and provides a new solution for CTF stripping.

## 2 Results and Discussion

### 2.1 Morphology and Structure

**Figure 1:**
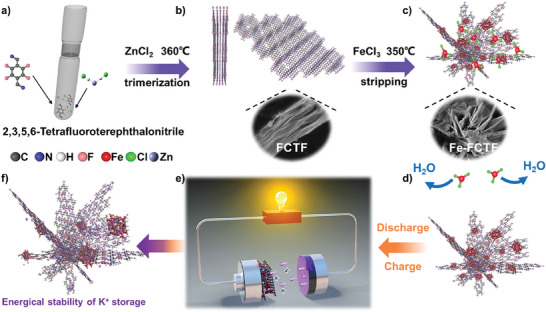
The schematic diagram of Fe@FCTF preparation and its application in PIBs.

**Figure** **1** illustrates the preparation, stripping, and utilization of the FCTF in PIBs. Initially, 2,3,5,6‐Tetrafluoroterephthalonitrile served as the precursor, and FCTF (Figure 1a,b) was synthesized by self‐polymerization via ZnCl_2_ catalysis. For salt stripping, FCTF and anhydrous FeCl_3_ were mixed in a weight ratio of 1:4 and maintained at 350 °C for 72 h. After removing the residual FeCl_3_ by washing, Fe@FCTFs were obtained (Figure 1c,d). A yield of 80.5% was obtained using this method, in Figure S1 (Supporting Information). It was anticipated that the fluoride within the FCTF would undergo substitution reactions with FeCl_3_, resulting in the formation of iron fluoride (FeF_3_) anchored onto the FCTF while simultaneously stripping the densely stacked FCTF. The stripped Fe@FCTF was expected to facilitate ion migration and diffusion, thus enhancing its K^+^ storage performance (Figure 1e,f). Notably, anchoring iron‐based clusters on FCTFs provides K^+^ storage active sites and further improves the K^+^ storage performance. For comparison, a fluorine‐free CTF and its stripped CTF (Fe@CTF) were synthesized via a similar process using 1,4‐Terephthalonitrile as the precursor (Figure S2, Supporting Information). As shown in Figure S3 (Supporting Information), the yield of Fe@CTF reached 77.8% after repeated washing and drying.

**Figure 2:**
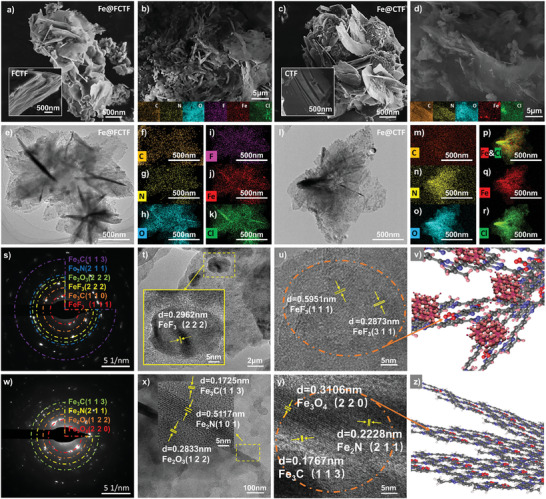
a) SEM images of Fe@FCTF and FCTF. b) EDS mapping of Fe@FCTF. c) SEM images of Fe@CTF and CTF. d) EDS mapping of Fe@CTF. e) Low‐resolution TEM image of Fe@FCTF. f–k) EDS elemental mappings of Fe@FCTF. l) Low‐resolution TEM image of Fe@CTF. (m‐r) EDS elemental mappings of Fe@CTF. s) SEAD pattern of Fe@FCTF. t–u) High‐resolution TEM image of Fe@FCTF. v) The schematic diagram of Fe@FCTF. w) SEAD pattern of Fe@CTF. x,y) High‐resolution TEM image of Fe@CTF. z) The schematic diagram of Fe@CTF.

**Figure** **2**a shows the SEM images of the Fe@FCTF and FCTF. This demonstrates that the Fe@FCTF exhibits loosely stacked, warped, layered structures distinct from the tightly stacked FCTF. Moreover, some layered structures were broken, indicating that the molten salt of the FeCl_3_ stripping process could not only achieve stripping of the laminated structure but also destroy the layered structure. This phenomenon may be attributed to the displacement reaction between FeCl_3_ and the FCTF, leading to the release of chlorine vapor bodies, which in turn caused swelling and peeling of the FCTF lamellae. In addition, SEM‐EDS elemental distribution scanning analysis revealed that the Fe@FCTF contained C, N, O, F, Fe, and Cl. The shape distribution of the Fe element was basically the same as that of the other elements, indicating that Fe was uniformly loaded onto the FCTF skeleton (Figure 2b). Point scans for localized areas rich in Fe showed that the weight percentages of these elements were 10.88%, 0.82%, 75.3%, 0.31%, 3.52%, and 9.18%, respectively (Figure S4, Supporting Information). Fe is a newly introduced element in Fe@FCTF, and the higher oxygen content may originate from Fe_2_O_3_, which is produced by oxidation during the sealing tube transfer process. In addition, due to the introduction of Fe, it may react chemically with N atoms to form Fe‐N compounds. Such a reaction may lead to a decrease in the free N content of the material (Figure S5, Supporting Information). To examine the thickness of the sheets among the as‐prepared samples, atomic force microscopy (AFM) was further employed. The specific number of layers and size distribution of the obtained Fe@FCTF nanosheets were evaluated using AFM (Figure S6, Supporting Information). The apparent AFM heights of most of the Fe@FCTF nanosheets range from 1.5 to 1.8 nm, indicating that the layer numbers of most Fe@FCTF nanosheets had two to three layers. In addition, an unpeeled FCTF was tested. The results show a large, irregularly connected, thick layer with a large lateral average monolith size. As shown in Figure S7 (Supporting Information), the apparent AFM average heights of the FCTF were 2.1 nm. Therefore, most FCTFs have five layers. This demonstrates the feasibility of the molten‐salt stripping strategy.^[^ ^54^, ^55^, ^56^ ^]^

The stripping behavior of fluorine‐free CTF in the molten FeCl_3_ was also carried out to verify the role of F in the stripping process. Figure 2c shows an SEM image of Fe@CTF, illustrating the exfoliation effect introduced by FeCl_3_ on the CTF. However, the exfoliation of the CTF was mainly limited to the surface, and the internal layered structure was preserved (Figure S8, Supporting Information). This indicates that F plays a crucial role in facilitating CTF stripping. In addition, elemental scanning analysis showed that the Fe@CTF contained C, N, O, Fe, and Cl. Notably, the elemental distribution map reveals bright red spots corresponding to Fe, indicating the attachment of large Fe‐based particles to the surface of the CTF. This distribution differs from the uniform loading of Fe observed in Fe@FCTF (Figure 2d; Figure S9, Supporting Information), suggesting that the presence of F facilitates the uniform loading of Fe onto the FCTF. At the same time, we can observe that the amount of N is significantly lower after stripping compared to the amount of N in the CTF before stripping. This phenomenon is similar to that observed in Fe@FCTF. This suggests that the FeCl_3_ stripping strategy can not only widen the interlayer spacing, but also form Fe‐N compounds on the surface of the CTF backbone to provide more active sites.

Figure 2e shows a Transmission Electron Microscope (TEM) image of Fe@FCTF with a lamellar morphology, where the CTF is connected in the middle and blown out in all directions, giving it a “petal‐like” stacked lamellae structure, differentiating it from the dense stacking FCTF (Figure S10, Supporting Information). The edges of the layers appear to be relatively transparent and untidy, suggesting that the exfoliated Fe@FCTF layers are thin and that the torn edges found in some regions of the fluffy structure may have been caused by the introduction of F. TEM with Energy Dispersive Spectroscopy (EDS) elemental scanning distribution analysis confirmed that Fe@FCTF contained C, N, O, F, Fe, and Cl. The newly introduced Fe was uniformly loaded onto the FCTF (Figure 2f–k), consistent with the SEM‐EDS analysis. To further investigate the role of F in the molten salt exfoliation process, the Fe@CTF was observed. Figure 2l shows that most of the Fe@CTF maintained a stacked layer resembling an unbloomed flower bud with only unidirectional extension. The elemental distribution diagram also shows five elements (C, N, O, Fe, and Cl) in the flake Fe@CTF material (Figure 2m–r).

The ring structure of the selected area electron diffraction (SAED) pattern (Figure 2s) reveals the polycrystalline structure of Fe@FCTF. SAED pattern analysis showed six distinct rings with d‐spacings corresponding to the (111) and (222) planes of FeF_3_, (113) and (110) planes of Fe_3_C, (211) planes of Fe_2_N, and (222) planes of Fe_2_O_3_.^[^ ^57^ ^]^ Upon inspecting the spotted area at high magnification, lattice fringes with spacings of 0.2962, 0.5951, and 0.2873 nm were observed, corresponding to the (222), (111), and (311) atomic planes of FeF_3_ (Figure 2t,u; Figure S11a, Supporting Information).^[^ ^58^, ^59^ ^]^ Furthermore, a lattice stripe with a pitch of 0.2271 nm was observed, corresponding to the (211) atomic plane of Fe_2_N. The lattice stripe with a planar spacing of 0.3473 nm corresponds to the (311) atomic plane of Fe_3_C, whereas the lattice stripe with a planar spacing of 0.8041 nm corresponds to the (010) atomic plane of C (Figure S11a, Supporting Information). Figure 2v shows a schematic of the structure, which may correspond to the material shown in Figure 2u.

As shown in Figure 2w, the blurred and discontinuous ring diffraction patterns indicate the polycrystalline nature of the Fe@CTF. The SAED patterns were indexed to the (113) plane of Fe_3_C, (211) plane of Fe_2_N, (122) plane of Fe_2_O_3_, and (220) plane of Fe_3_O_4_ based on their d‐spacings. As shown in Figure 2x, Figure 2y, and Figure S11b (Supporting Information), the clear stripes detected around the lamellae with lattice spacings may correspond to the (101) and (211) atomic planes of Fe_2_N and the (113) and (110) atomic planes of Fe_3_C, respectively, which correspond to the above calibration results. Figure 2z is a schematic representation of a structure that may correspond to the material in Figure 2y. This is similar to the phenomenon observed after Fe@FCTF stripping. In contrast, large amounts of Fe_2_O_3_ and Fe_3_O_4_ are detected at the edges. This indicates that, without the help of F, most of the Fe floats on the surface of the material in the oxidized form, and a small amount of Fe can be firmly anchored to the lamellae. Therefore, uneven bright red spots are observed in the elemental distribution map, consistent with the SEM results.^[^ ^60^ ^]^ However, no obvious lattice fringes were observed in the FCTF/CTF before exfoliation, indicating an amorphous structure (Figures S12 and S13, Supporting Information).

**Figure 3:**
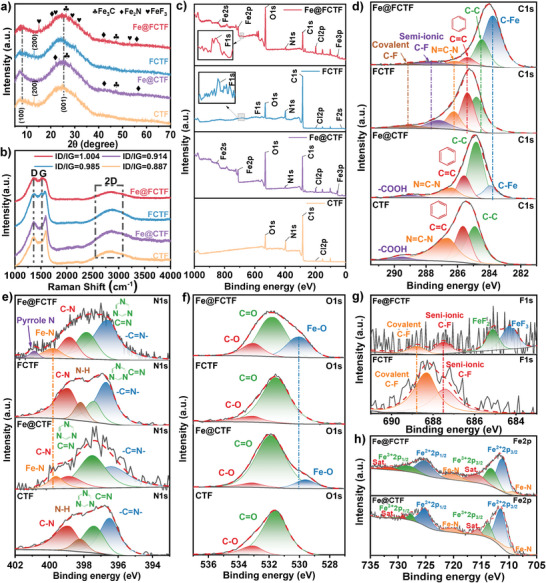
a) XRD patterns of Fe@FCTF (red), FCTF (blue), Fe@CTF (purple), and CTF (yellow). b) Raman spectrum. c–h) XPS spectra of Fe@FCTF and other CTFs: c) survey, d) C1s, e) N1s, f) O1s, g) F1s, and (h) Fe2p.

The powder XRD pattern of Fe@FCTF is illustrated in **Figure** **3**a, along with those of the three other samples: fluorinated FCTF, exfoliated non‐fluorinated Fe@CTF, and pristine CTF. In the spectrum of the Fe@FCTF, two prominent diffraction peaks at 6° and 23° are close to the typical FCTF, corresponding to the (100) and (001) planes, respectively. This suggests that the thin‐layer structure of Fe@FCTF maintained the molecular stacking pattern of the FCTF.^[^ ^61^, ^62^, ^63^, ^64^ ^]^ Similarly, the Fe@CTF and CTF exhibited two broad peaks at 6° and 23°, indicating the persistence of the 2D stacking structure despite FeCl_3_ stripping and thinning. In a comparative analysis, the weak peaks at 14.82°, 23.91°, 28.69°, 30.1°, and 52.84° correspond to the (111), (220), (311), (222), and (531) planar reflections of FeF_3_, respectively, with corresponding interplanar distances of 0.594, 0.371, 0.311, 0.296, and 0.173 nm, respectively, which match the interplanar distances of FeF_3_ calibrated during TEM observation (Figure 2t,u). The 26.47° and 48.74° peaks correspond to the reflections of Fe_3_C planes (110) and (113), respectively. The peaks at 20.42°, 37.21°, 42.97°, and 56.72° correspond to the reflections in the Fe_2_N plane (001), (020), (211), and (022) planes, respectively.^[^ ^65^, ^66^ ^]^ Similarly, in the Fe@CTF spectra, the peaks correspond to Fe_3_C at 26.47° and 48.74°, and the peaks correspond to F_2_N at 20.21°, 42.61°, and 56.57°.

Figure 3b shows the Raman spectrum of the Fe@FCTF compared to those of the FCTF, Fe@CTF, and CTF. The Fe@FCTF spectrum displayed three distinct peaks at 1362, 1511, and 2869 cm^−1^, corresponding to the D, G, and 2D peaks. The D peak is attributed to the defect vibration of the carbon atoms, and the G peak is attributed to the ordered vibration of the carbon atoms in the plane. The integrated intensity ratios (ID/IG) of the D and G peaks were used to evaluate the structural integrity of the materials.^[^ ^3^, ^67^ ^]^ The ID/IG ratio of the Fe@FCTF was 1.004, which was higher than that of the FCTF (0.985). Similarly, the ID/IG ratio of Fe@CTF was 0.914, higher than that of the CTF (0.887). This indicates that the stripping of FeCl_3_ leads to a certain degree of disruption in the structure of the FCTF and CTF, as evidenced by the SEM observations of the torn lamellar structures, intermediate connections, and edge defects (Figure 2b,d).

Fourier transform infrared (FTIR) spectroscopy was performed to investigate the organic functionalities and bonding frameworks in the CTFs. The FTIR spectral bands of Fe@FCTF, FCTF, Fe@CTF, and CTF samples between 4000 to 600 cm^−1^ are shown in Figure S14 (Supporting Information). In Fe@FCTF and Fe@CTF, a broad absorption peak at 3460 cm^−1^ was observed. This peak resulted from the superposition of the ‐O‐H stretching vibration absorption peak and the ‐N‐H stretching vibration absorption peak of the groups involved in hydrogen bonding, causing it to widen.^[^ ^68^, ^69^, ^70^ ^]^ The peaks at ≈1386 cm^−1^ and 1590 cm^−1^ reveal the existence of triazine and benzene rings.^[^ ^71^, ^72^ ^]^ As we know, the electron‐absorbing base moves the infrared absorption peak to the high‐frequency region, and the electron‐supplying base moves the infrared absorption peak to the low‐frequency region.^[^ ^69^, ^73^, ^74^ ^]^ The two characteristic peaks at 1386 cm^−1^ and 1590 cm^−1^ were attributed to the stretching vibrations of ─C─N═and ─C═N─ on the triazine ring, respectively. This indicated that the stripping process did not cause serious damage to the chemical structure of the samples, allowing the triazine ring structure to be preserved. Specifically, the absorption peaks of the stripped samples are shifted toward higher frequencies compared to those of the FCTF and CTF, which may be attributed to the formation of Fe complexes. Finally, the band at 773 cm^−1^ is associated with the out‐of‐plane bending of the C‐H group in the monosubstituted aromatic rings.

In addition, the porous properties and specific areas of the CTFs materials were characterized by N_2_‐adsorption‐desorption isothermal analysis (BET), in Figure S15a,b (Supporting Information). The N_2_ adsorption/desorption isotherms of the Fe@FCTF and FCTF can be classified as typical Type I, indicating the microporous nature of the material.^[^ ^75^, ^76^, ^77^ ^]^ Using the Brunauer‐Emmett‐Teller formula, the specific surface area of Fe@FCTF was calculated to be 450.6 m^2^ g^−1^, which is approximately 68 m^2^ g^−1^ higher than that of the FCTF (383.1 m^2^ g^−1^). This implies that a decrease in the number of stacked layers in the CTFs increases their BET‐specific surface area. Nonlocal density functional theory (NL‐DFT)‐derived pore size distribution curve shows the presence of micropores ≈3.1 nm and 2.4 nm in the skeletons of Fe@FCTF and FCTF, respectively. There was a slight increase in pore size after stripping.

The N_2_ adsorption/desorption isotherms of Fe@CTF and CTF (Figure S15c,d, Supporting Information) show the combined characteristics of type I and type IV, indicating microporous and mesoporous structures.^[^ ^78^, ^79^ ^]^ Compared to that of the CTF (336.7 m^2^ g^−1^), the exfoliated Fe@CTF has a smaller surface area of 109.3 m^2^ g^−1^, which should be ascribed to the poor 2D layered stacking status of the Fe@CTF. The resultant random overlaid few‐layered 2D structure after exfoliation causes some existing pores/channels to be covered or sealed by randomly stacked layers to some extent, which cannot be detected by N_2_ adsorption/desorption analysis. Such a phenomenon (smaller BET surface area after exfoliation) has also been detected in several previous reports of exfoliated few‐layered COF materials.^[^ ^72^, ^75^, ^77^ ^]^ In addition, the average pore size of Fe@CTF increased significantly to 5.2 nm compared to the average pore size of the CTF before stripping (2.1 nm). The test results were recorded in tabular form (Table S1, Supporting Information).

The thermal stabilities of the Fe@FCTF and FCTF were examined by thermogravimetric analysis (TGA) from 30–1000 °C under an Ar atmosphere, as illustrated in Figure S16 (Supporting Information). The Fe@FCTF exhibited a three‐step weight loss. The weight loss of 7.1% in the first stage was due to water adsorption from the Fe@FCTF. The second stage of weight loss occurs between 370 and 700 °C.^[^ ^80^, ^81^ ^]^ For the Fe@FCTF, the mass loss at this stage was due to the decomposition of organic iron and the framework. The third stage occurs after 800 °C, and the mass loss in this stage may be related to the decomposition of inorganic iron and the formation of iron oxide. However, the FCTF exhibited a two‐step weight loss. The first very small weight loss (10%) step in the TGA curve below 120 °C was attributed to the loss of water. The second rapid major mass loss step at around 300 °C was attributed to the breakdown of the framework. As the temperature increased, the aromatic structure began to carbonize, and the molecular structure was further destroyed and decomposed.^[^ ^80^, ^82^ ^]^ The residual mass of FCTF after heating at 1000 °C was calculated to be 60%. As shown in Figure S17 (Supporting Information), the CTFs were mixed with powdered poltvinylidenefluoride (PVDF) at 4:1 to produce homogeneous thin discs. The conductivity of CTFs was tested using the four‐probe method and obtained as 2.35 × 10^−4^, 1.94 × 10^−4^, 2.1 × 10^−4^, and 1.88 × 10^−4^ s cm^−1^ for Fe@FCTF, FCTF, Fe@CTF, and CTF, respectively. The electrical conductivity of the composites after exfoliation was significantly better than before, which can be attributed to the introduction of some Fe. The test results were recorded in tabular form (Tables S2‐S5, Supporting Information).

The chemical composition of Fe@CTF, FCTF, Fe@CTF, and CTF surfaces was analyzed using XPS. The analysis revealed that the Fe@FCTF surface contained Fe, F, O, N, C, and Cl, with characteristic peaks at 284, 399, 532, 686, and 710 eV, respectively (Figure 3c). The content ratios of these elements were 30.41%, 16.66%, 23.62%, 9.97%, 12.79%, and 6.55%, respectively (Table S6, Supporting Information). In contrast, the pristine FCTF surface contains only F, O, N, C, and Cl. This indicates that the anchoring of Fe on the FCTF structure was accomplished through the molten salt exfoliation of FeCl_3_. The measured spectrum in Figure 3c confirms the presence of C, N, O, Fe, and Cl in the Fe@CTF, whereas the pristine CTF only contained C, N, O, and Cl. The atomic percentages on the sample surface showed that the percentage of Fe in Fe@FCTF was approximately twice that in Fe@CTF, indicating that the introduction of F assisted in anchoring Fe onto the surface of the material (Table S7, Supporting Information).

To investigate the anchoring forms of the Fe and FCTF structures, the C1s, N1s, O1s, F1s, Fe2p, and Cl2p were analyzed. The results showed that the C1s peak of the Fe@FCTF shifted to the right compared with that of the original FCTF. This shift can be resolved into six subpeaks: covalent C‐F (289.3 eV), semi‐ionic C‐F (287.84 eV), triazine ring‐connected C═N (286.2 eV), C═C (285.6 eV), ‐d C‐C (284.5 eV) attached to a benzene ring, and C‐Fe (283.7 eV). In contrast, the analysis of C1s in the FCTF only revealed covalent C‐F (289.1 eV), semi‐ionic C‐F (287.17 eV), C═N (286.2 eV) connected to the triazine ring, C═C (285.35 eV) connected to the benzene ring, and C‐C (284.7 eV), without any peak corresponding to C‐Fe (Figure 3d).^[^ ^83^ ^]^The C1s peak of the Fe@CTF shifted significantly to the right compared to that of the original CTF, as shown in Figure 3d. The Fe@CTF peak can be resolved into five sub‐peaks: the carboxyl group (COOH) (289.1 eV), C═N (286.38 eV), C═C (285.67 eV), C‐C (284.82 eV) attached to a benzene ring, and C‐Fe (283.96 eV). However, in the C1s analysis of the CTF, only carboxyl COOH (289.54 eV), C═N (286.6 eV) connected to the triazine ring, C═C (285.65 eV), and C‐C (284.91 eV) connected to the benzene ring were observed. No subpeaks corresponding to C‐Fe were observed.^[^ ^84^ ^]^ This study confirmed that during the stripping process of FeCl_3_, Fe can be anchored to the carbon atom skeleton of the CTF. The proportion of C‐Fe was significantly lower (45.21%) than Fe@FCTF (Table S7, Supporting Information).

The results of the N1s spectrum (Figure 3e) showed that in Fe@FCTF, the peaks at 400, 399.6, 398.4, 397.3, and 396 eV are attributed to pyrrole nitrogen, N‐coordinated Fe, pyridine nitrogen, and triazine‐linked Fe@FCTF, respectively.^[^ ^85^ ^]^ Additionally, pyrrole‐nitrogen and pyridinium‐nitrogen can improve the surface charge distribution of carbon flakes and cause localized electron defects, thus facilitating ion diffusion kinetics and potassium storage through surface adsorption. The O1s spectrum (Figure 3f) demonstrated that Fe@FCTF had a Fe‐O bond at 529.9 eV, indicating the partial oxidation of Fe, which was consistent with the TEM analysis. The figure also revealed a higher binding energy for the Fe‐O bonds in Fe@FCTF, an observation that can be attributed to the chemical environment surrounding the Fe atoms in this material. In Fe@FCTF, the Fe atoms were found to bind to F and N, elements that possess higher electronegativities than oxygen. The greater electronegativity of F and N atoms inherently results in a stronger attraction for electrons, thereby enhancing the covalency of the Fe‐F and Fe‐N bonds. This increased covalency, in turn, leads to a relatively stronger binding energy for the Fe‐O bonds in Fe@FCTF compared to those in Fe@CTF. The Cl2p at 199.4 eV and 197.6 eV in Fe@FCTF can be attributed to the C‐Cl bond and N‐Cl bond, respectively, while the Cl2p at 198.9 eV can be attributed to Fe‐Cl. These findings suggest that a portion of Fe^3+^ was reduced to Fe^2+^ and combined with Cl, which remained in the sample (Figure S18, Supporting Information). As shown in Figure 3g, introducing Fe into the Fe@FCTF caused a significant right shift in the F1s spectrum compared to the original FCTF. The spectrum can be divided into four peaks corresponding to covalent C‐F (688.8 eV), semi‐ionic C‐F (687.5 eV), FeF_2_ (685.5 eV), and FeF_3_ (684.3 eV), whereas the F1s spectrum of the original FCTF has only two peaks. According to Table S7 (Supporting Information), the proportions of C‐F after stripping were 2.04% and 5.04%, significantly lower than the 5.1% and 11.6% before the reaction, respectively. This indicates that part of the C‐F was transformed into C‐Fe during the stripping process of FeCl_3_, thus anchoring it to the carbon‐atom backbone of the FCTF. Further analysis of Fe2p (Figure 3h) showed that the peaks of Fe2p_3/2_ and Fe2p_1/2_ were mainly concentrated at 711.9, 713.12, 725.08, 727.98, 710.8 eV (Fe2p_3/2_) and 720.01 eV (Fe2p_1/2_), respectively, which can be assigned to N‐coordinated Fe^2+^ and Fe^3+^ species, iron carbides, and iron oxides.^[^ ^86^ ^]^ The satellite peaks at 715.49 eV and 731.12 eV were auxiliary, indicating the coexistence of FeII and FeIII, corresponding to FeF_3_, FeF_2_, Fe_2_N, Fe_3_C, and Fe_2_O_3_. The Fe2p_3/2_ spectrum in Fe@CTF can be deconvoluted into a main peak at 709.36 eV and a small peak at 720.01 eV, corresponding to N‐coordinated Fe^2+^ and Fe^3+^ species. The proportions of these species were smaller in Fe@FCTF, indicating that fluorination promoted the binding of Fe to N (Table S8, Supporting Information). Additionally, the presence of satellite peaks at 727.65 eV and 713.8 eV in the Fe2p_3/2_ spectrum suggests the presence of Fe^2+^ and Fe^3+^ on the surface of the material. These peaks correspond to Fe_3_C, Fe_2_N, Fe_2_O_3_, or Fe_3_O_4_.^[^ ^84^ ^]^

### 2.2 Electrochemical Performance

**Figure 4:**
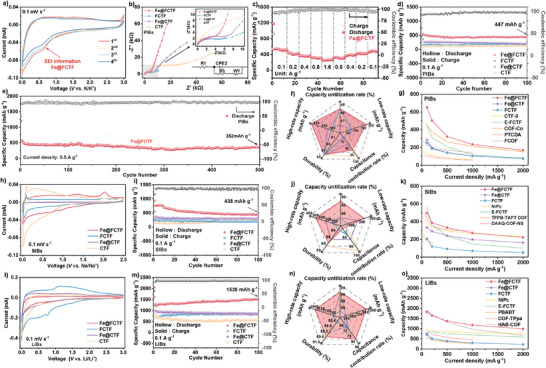
a) CV curves of Fe@FCTF at first four cycles at 0.1 mV s^−1^ for PIBs. b) The Nyquist plots for the Fe@FCTF and other CTFs for PIBs. c) Rate capability of Fe@FCTF from 0.1‐1.6 A g^−1^ for PIBs. d) Cycling performance of Fe@FCTF, and other CTFs at 0.1 A g^−1^ for PIBs. e) Long cycling stability of the Fe@FCTF electrode at 0.5 A g^−1^ for PIBs. f) In PIBs: radar chart of the performances for Fe@FCTF (red curve), FCTF (blue curve), Fe@CTF (purple curve), and CTF (yellow curve). g) Rate capability comparison of Fe@FCTF, Fe@CTF, FCTF electrode, and reported anodes for PIBs. h) CV curves of Fe@FCTF and other CTFs at first four cycles at 0.1 mV s^−1^ for SIBs. i) Cycling performance of Fe@FCTF, and other CTFs at 0.1 A g^−1^ for SIBs. j) In SIBs: radar chart of the performances for Fe@FCTF (red curve), FCTF (blue curve), Fe@CTF (purple curve), and CTF (yellow curve). k) Rate capability comparison of Fe@FCTF, Fe@CTF, FCTF electrode, and reported anodes for SIBs. l) CV curves of Fe@FCTF and other CTFs at first four cycles at 0.1 mV s^−1^ for LIBs. m) Cycling performance of Fe@FCTF, and other CTFs at 0.1 A g^−1^ for LIBs. n) In LIBs: radar chart of the performances for Fe@FCTF (red curve), FCTF (blue curve), Fe@CTF (purple curve), and CTF (yellow curve). o) Rate capability comparison of Fe@FCTF, Fe@CTF, FCTF electrode, and reported anodes for LIBs.

A cyclic voltammetry (CV) test was applied for the Fe@FCTF electrode (0.1 mV s^−1^ and 0.0–3.0 V) to investigate its potassium‐ion insertion/extraction behavior, **Figure** **4**a. The first cathodic scan of the Fe@FCTF contains one dominant cathodic peak at 0.6 V, which may be associated with the insertion of potassium ions into the triazine rings. This may also be due to the reaction of potassium ions with FeF_3_ and the generation of solid electrolyte interface (SEI) films. In the oxidation process, there is a corresponding anodic peak at 0.61 V. Comparatively, the weakened peaks at 0.57 V can be detected for the FCTF in the cathodic scan, indicating its less involvement of triazine rings and FeF_3_ in potassium storage. As shown in Figure 4b, the electrochemical impedance spectroscopy (EIS) of the cells with the Fe@FCTF, FCTF, Fe@CTF, and CTF electrodes were obtained. The AC impedance spectrum semicircle diameter of the stripped CTFs battery is significantly smaller than that of the CTFs battery before stripping, and the Fe@FCTF and Fe@CTF batteries are about 3.5 and 6 kΩ, respectively, while the FCTF and CTF batteries are ≈4.3 and 23 kΩ, respectively. This shows that the exfoliated CTFs have a better charge transfer ability.

Figures 4c and S19 (Supporting Information) display the rate performance and galvanostatic charge/discharge curves of Fe@FCTF at different rates. After the exfoliation treatment, Fe@FCTF exhibited large reversible specific capacities, which are 541 mAh g^−1^ at 0.1 A g^−1^, 437 mAh g^−1^ at 0.2 A g^−1^, 370 mAh g^−1^ at 0.4 A g^−1^, 323 mAh g^−1^ at 0.8 A g^−1^, and 257 mAh g^−1^ at 1.6 A g^−1^. When the test current was gradually restored to 0.8, 0.4, 0.2, and 0.1 A g^−1^, the reversible capacity recovered to 330, 378, 436, and 503 mAh g^−1^. Figures S20 and S21 (Supporting Information) show the rate performances of the Fe@FCTF and CTF. The discharge‐specific capacities of the Fe@FCTF were 653, 503, 324, 232, and 169 mAh g^−1^ at 0.1, 0.2, 0.5, 1, and 2 A g^−1^, respectively, reaching 560 mAh g^−1^ at a current density of 0.1 A g^−1^. However, the discharge‐specific capacity of the FCTF was only half that of the Fe@FCTF under the same test environment at 251, 186, 140, 103, and 74 mAh g^−1^. For comparison, Fe@CTF and CTF electrodes were tested under the same conditions, and the Fe@CTF electrodes exhibited discharge specific capacity of 286, 193, 142, 105, and 75 mAh g^−1^ at 0.1, 0.2, 0.5, 1, and 2 A g^−1^, respectively. The discharge‐specific capacity of the CTF electrodes varied and was 110, 98, 60, 46, and 33 mAh g^−1^. The better rate performance exhibited by the Fe@FCTF can be attributed to its divergent sheet structures.

Figures 4d and S22 (Supporting Information) show that at a current density of 0.1 A g^−1^, the initial efficiency/reduction ratio capacities of Fe@FCTF and FCTF are 1217/557 and 385/174 mAh g^−1^, respectively, and the Coulombic efficiency (CE) was less than 50%, which may be due to the effect of SEI formation. Based on the activation induced by the improved K diffusion kinetics during cycling, the reversible capacity of the Fe@FCTF gradually increased after a few cycles, and the Coulombic efficiency gradually recovered to approximately 100%. The stability of Coulombic efficiency remained consistent for 100 cycles with a capacity of 447 mAh g^−1^. In contrast, the FCTF exhibited a specific capacity of only 206 mAh g^−1^ under the same test conditions, which is half of that of the Fe@FCTF, which showed a stable capacity of 227 mAh g^−1^ (significantly lower than that of the Fe@FCTF), and the CTF showed a stable capacity of 144 mAh g^−1^. This may be due to the introduction of fluorine atoms, which assist in anchoring Fe and facilitate potassium storage in the Fe@FCTF. These results reveal the great advantage of the exfoliated covalent framework for storing the K^+^ energy of PIBs. Furthermore, the long‐term cycling performance of the batteries was verified. After 500 cycles, the Fe@FCTF electrode maintained a high capacity at 0.5 A g^−1^ of 352 mAh g^−1^. The CE was 100% during the entire cycle with only a small capacity decay, implying ultra‐stable performance, Figure 4e. Figure S23 (Supporting Information) shows a clear plateau at 0.6 V during the first lap of the discharge, corresponding to the oxidation peak at 0.6 V in the CV scan curve, indicating the extraction of potassium ions. After 1, 100, 200, and 500 cycles, the discharge‐charge curves of Fe@FCTF have similar shapes, and their reversible capacities are almost the same, indicating the good electrochemical reversibility of the exfoliated composites. These results reveal the great advantage of the exfoliated covalent framework for storing the K^+^ energy of PIBs. As shown in the radargram in Figure 4f, the comprehensive performance of the Fe@FCTF electrode in PIBs was superior to that of several other CTF electrodes. The summary plots in Figure 4g show that the Fe@FCTF electrodes applied to potassium anodes exhibit an impressive superior rate capability compared to reported COF materials such as CTF‐0, E‐FCTF, COF‐Co, PTCDA, and FCOF.^[^ ^27^, ^72^, ^87^, ^88^, ^89^ ^]^

The stripped Fe@FCTF and Fe@CTF also exhibited better electrochemical properties in the SIBs and LIBs than the FCTF and CTF. CV curves were measured for each sample in the potential range of 0 to 2.5 V (versus Na/Na^+^) at a scan rate of 0.1 mV s^−1^, Figure 4h. When charged and discharged at a slow rate of 0.1 mV s^−1^, the CV curves for the CTF samples follow a clear pattern that matches up well with the charging and discharging graphs. At the start of the discharge process, there are two small peaks at ≈0.6 V and 0.8 V. These peaks are usually caused by the breakdown of the electrolyte and the formation of a SEI layer on the surface of the electrode. The steep peaks close to 0.1 V and the gentle rises over a range of 0.2 to 1.5 V represent the flat and gradual parts of the charging and discharging graphs, respectively. As shown in Figure S24 (Supporting Information), the AC impedance spectrum of the CTFs battery after peeling has no apparent semicircle diameter, while the CTFs (FCTF, CTF) before peeling can detect Rct values of 43 and 62 Ω, respectively. Fe@FCTF exhibited excellent rate capability in SIBs and maintained discharge‐specific capacities of 329 and 68 mAh g^−1^ at 0.1 A g^−1^ and 2 A g^−1^, which were the best results for all CTFs electrode rate capabilities, Figures S25 and S26 (Supporting Information). In addition, the Fe@FCTF and Fe@CTF electrodes exhibited higher first‐cycle sodium extraction capacities of 766 and 556 mAh g^−1^ at 0.1 A g^−1^, while the FCTF and CTF electrodes exhibited specific capacities of 287 and 154 mAh g^−1^, respectively (Figure 4i). Figure S27 (Supporting Information) shows an apparent plateau at 0.6 V during the first lap of the discharge, corresponding to the oxidation peak at 0.6 V in the CV scan curve, indicating the extraction of sodium ions. For the Fe@FCTF anode, a specific capacity of 438 mAh g^−1^ can be observed after 100 cycles, showing a capacity fading similar to that in potassium‐ion batteries, which may be due to the larger ionic radius of Na^+^, leading to the formation of SEI, and slow ion movement. It may also be that some sodium ions are only adsorbed on the surface of Fe@FCTF, which may have poor reversibility, resulting in a capacity drop in the first few cycles. Many previous studies have demonstrated this phenomenon (stable in LIBs but not in other alkali‐ion batteries).^[^ ^90^, ^91^ ^]^ As shown in the radar diagram in Figure 4j, the comprehensive performance of the Fe@FCTF electrodes in SIBs is superior to that of several other CTF electrodes. The rate capability of the Fe@FCTF electrode exceeds that of most reported COF electrodes in SIBs anodes (e.g., NiPc, E‐FCTF, DAAQ‐COF‐NS, and TFPB‐TAPT‐COF), as shown in Figure 4k.^[^ ^72^ ^,^ ^92^, ^93^, ^94^, ^95^, ^96^ ^]^

Using Fe@FCTF as the anode of LIBs, CV curves were measured for each sample in the potential range of 0.0–3.0 V (versus Li/Li^+^) at a scan rate of 0.1 mV s^−1^, Figure 4l. It can be seen that FCTF, Fe@CTF, and CTF have well‐resolved redox peaks compared with that of Fe@FCTF, which includes two oxidation peaks (charging/insertion) during the intercalation process and two reduction peaks (discharging/extraction) during the de‐intercalation process. According to the Nyquist plot (Figure S28, Supporting Information), the charge transport resistance of Fe@FCTF is as low as 260 Ω. These results were minimal with respect to the other CTFs. The Fe@FCTF electrode exhibited an excellent rate performance (1832/992 mAh g^−1^ at 0.1 A g^−1^/2 A g^−1^), as shown in Figures S29 and S30 (Supporting Information). Even when the test current returns to 0.1 A g^−1^, the capacity remains as high as 1785 mAh g^−1^. In addition, a discharge‐ and charge‐specific capacity of 2054/1246 mAh g^−1^ was obtained in the first cycle of the Fe@FCTF, with a CE value of 60.7%, which was better than that of the FCTF electrode (Figure 4m; Figure S31, Supporting Information). After 100 cycles, the reversible capacity of Fe@FCTF was still as high as 1526 mAh g^−1^, the retention rate was close to 100%, and there was an upward trend, which could be attributed to the even attachment of the FeF_3_ redox sites on the sheet.^[^ ^90^ ^]^ As shown in the radargram in Figure 4n, the comprehensive performance of the Fe@FCTF electrode in LIBs was superior to that of several other CTF electrodes. Notably, the stable specific capacity of Fe@FCTF at a low current was higher than that of reported organic anodes for LIBs, Figure 4o.^[^ ^72^ ^,^ ^97^, ^98^, ^99^, ^100^, ^101^, ^102^, ^103^ ^]^

In addition, to further explore the electrochemical stability of the stripped Fe@FCTF in alkali metal batteries, long‐term cycle stability tests of constant‐current charging and discharging were performed in different batteries. When Fe@FCTF was used as the anode of the SIBs, its specific capacity tended to increase at a current density of 0.5 A g^−1^. Simultaneously, we observed the CV curve of the first cycle at a low scan rate (Figure S32a, Supporting Information), which appeared at ≈1.2 V. The wider oxidation peak can be attributed to the formation of the SEI film; the impact of sodium ions destroyed part of the material structure, the last three circles tended to overlap, and the material structure was fixed. After 500 cycles, Fe@FCTF stabilized at a large specific capacity of 357 mAh g^−1^ in SIBs, surpassing most of the currently reported organic anodes for SIBs. As shown in Figure S32b (Supporting Information), at a current density of 0.5 A g^−1^, the Fe@FCTF electrode applied to the anode of a LIB still exhibited a large specific capacity of 988 mAh g^−1^ after 500 cycles. At a scan rate of 0.1 mV s^−1^, the CV curves of the first four cycles did not show any obvious SEI film formation information, indicating that the stripped Fe@FCTF provided a wider ion transport channel for lithium ions with a smaller radius, effectively reducing the loss of lithium. These results reveal the universal nature of Fe@FCTF for anode alkali‐ion storage in secondary batteries.

**Figure 5:**
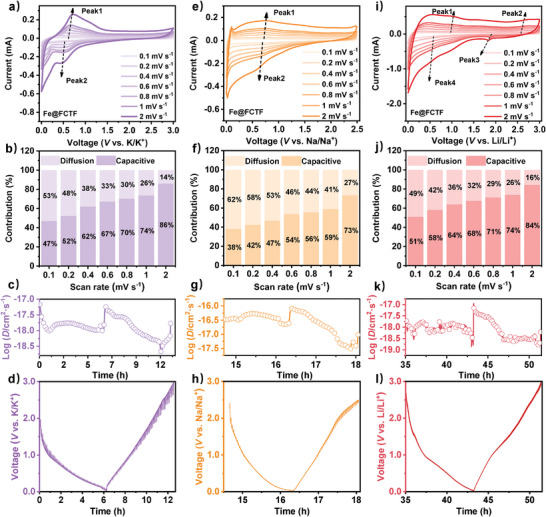
PIBs: a) CV plots of Fe@FCTF with different scan rates of 0.1 to 2 mV s^−1^. b) Contribution ratio of capacitive and diffusion‐controlled behaviors of Fe@FCTF at various scan rates. c) The calculated K^+^ chemical diffusion coefficients for Fe@FCTF. d) GITT curve of Fe@FCTF electrode. SIBs: e) CV plots of Fe@FCTF with different scan rates of 0.1 to 2 mV s^−1^. f) Contribution ratio of capacitive and diffusion‐controlled behaviors of Fe@FCTF at various scan rates. g) The calculated Na^+^ chemical diffusion coefficients for Fe@FCTF. (h) GITT curve of Fe@FCTF electrode. LIBs: i) CV plots of Fe@FCTF with different scan rates of 0.1 to 2 mV s^−1^. j) Contribution ratio of capacitive and diffusion‐controlled behaviors of Fe@FCTF at various scan rates. k) The calculated Li^+^ chemical diffusion coefficients for Fe@FCTF. (l) GITT curve of Fe@FCTF electrode.

CV tests were performed for Fe@FCTF and other CTFs at scan rates of 0.1, 0.2, 0.4, 0.6, 0.8, 1, and 2 mV s^−1^ in a potential window of 0.0–3.0 V to further explore the K^+^ storage behavior, **Figures** **5**a and S33 (Supporting Information). The results showed that the current intensity increased with increasing scan rate. The different curves have similar shapes, indicating that the Fe@FCTF has similar electrochemical properties at different scan rates, confirming its stable electrochemical properties. Notably, at a given potential, the peak current (i) was not proportional to the square root of the scan rate (v). This suggests that the redox process at the electrode was not merely ion diffusion controlled. In general, the corresponding peak current varied with the scan rate.

(1) $$i_{\left(v\right)}=av^{b}$$

(2) $$\log\;i_{\left(v\right)}=\log\;a+\mathrm{blog}\;v$$

From the reported studies, log(i) and log(v) can be fitted linearly to obtain the parameter b. This parameter determines the pseudocapacitive behavior during charge and discharge cycles. When *b* was 0.5, diffusion, such as the insertion/extraction between layers, controlled the reaction. This suggests that the electrode material has battery properties. When b is greater than or equal to 1, it implies that i(v) = av, indicating that the surface controls reactions such as surface adsorption. As shown in Figure S34 (Supporting Information), the two calculated *b*‐values of the anodic (1.056) and cathodic (0.889) peaks of Fe@FCTF were significantly higher than those of the FCTF (0.671 and 0.729, respectively), confirming the apparent pseudocapacitive contribution. In addition, the charge‐storage contribution rate was quantitatively calculated using the Dunn method.

(3) $$i_{\left(V\right)}=i_{\mathrm{cap}}+i_{\mathrm{diff}}=k_{1}v+k_{2}v^{1/2}$$

where i represents the current response, divided into capacitive‐controlled (K_1_) and diffusion (K_2_) controlled processes at a fixed voltage. As the scan rate increased, the proportions of the capacitive contributions of both the Fe@FCTF and FCTF increased significantly (Figure 5b; Figure S35, Supporting Information). Under the same experimental conditions, compared with the total current, the proportion of the Fe@FCTF electrode material on the voltage curve of the capacitive current at 2 mV s^−1^ was 86%, and that of the FCTF electrode was lower (67%), indicating that the exfoliated Fe@FCTF electrode has faster potassium reaction kinetics. This study used the galvanostatic intermittent titration technique (GITT) to evaluate the potassium ions' diffusion coefficients in the Fe@FCTF electrode (Figure 5c,d; Figure S36, Supporting Information). The GITT results showed that the diffusion coefficient of K^+^ between the electrolyte and the electrode followed Fick's second law, and the diffusion coefficient D value of the Fe@FCTF electrode (10^−17^‐10^−18^ cm^2^ s^−1^) was significantly higher than that of the non‐stripped FCTF electrode (10^−18^‐10^−19^ cm^2^ s^−1^), indicating shortening of the ionic diffusion paths of the electrode and an increase in the ionic mobility after stripping. As shown in Figure S37 (Supporting Information), it is easy to find that the contribution of the pseudo part is overwhelming. The calculated b‐values of the anodic (0.852, 0.841) and cathodic (0.933) peaks of Fe@CTF and the b‐values of CTF (1.316, 1.46, and 1.333) indicated that they were mainly controlled by surface adsorption. In all cases, the capacitive contributions of the exfoliated Fe@FCTF and Fe@CTF electrode materials can be attributed to their multi‐directional extended sheet structures and the FeF_3_ clusters uniformly attached to the sheet surface, which can provide abundant pathways for fast charge transfer and improved long‐term cycle life.

To further investigate the charge storage mechanism of the Fe@FCTF electrode, the CV curves measured at scan rates ranging from 0.1 to 2 mV s^−1^ in SIBs and LIBs are shown. In SIBs, as shown in Figures 5e and S38 (Supporting Information), two peaks of Fe@FCTF are selected for kinetic analysis: a broad peak at 0.63 V and a camel hump at 0.74 V, both of which correspond to the slope region above 0.6 V in the charge‐discharge curve. As shown in Figure S39 (Supporting Information), the log (i) curves of the two peaks in the Fe@FCTF exhibit a good linear relationship. For the broad peak at 0.63 V, the fitted b value is 0.823 (close to 1), and for the peak at 0.74 V, the fitted b value is 0.829 (close to 1), indicating that the entire process is mainly non‐diffusion‐limited. This result also supports the hypothesis that slope capacity originates from the pseudo‐adsorption of Na^+^. According to the fitting results, the charge storage mechanism of all the CTFs used in SIBs was mainly adsorption. Dunn analysis was then used to quantitatively separate the diffusion‐controlled and surface capacitance contributions, giving 38% and 51% diffusion‐controlled capacitance for Fe@FCTF and FCTF electrodes at a scan rate of 0.1 mV s^−1^ contribution, which gradually increased to 73% and 80% as the scan rate reached 2 mV s^−1^, Figures 5f and S40 (Supporting Information). The Na diffusion kinetics of the Fe@FCTF electrode were further investigated (Figure 5g,h), and the FCTF electrode was tested under the same test conditions (Figure S41, Supporting Information). The results indicated that The D values (10^−16^‐10^−17^ cm^2^ s^−1^) of the Fe@FCTF electrode are higher than those of the unstripped FCTF electrode (10^−17^‐10^−18^ cm^2^ s^−1^). This further confirmed that the peel‐and‐insert layering strategy used in this study facilitated the diffusive migration of large ions. A similar situation occurred for the unstripped Fe@CTF and CTF electrodes, with diffusion‐controlled capacitance contributions of 37%, 52%, 85%, and 98% at 0.1 mV s^−1^ and 2 mV s^−1^, respectively (Figure S42, Supporting Information).

In LIBs, as shown in Figures 5i and S43 (Supporting Information), the shape of the curve is similar, again confirming stable electrochemical performance. The broad peaks at 0.9, 2.69, 1.89, and 0.54 V. It can be seen that the b value of the anode (b_peak1_ = 0.868, b_peak2_ = 0.971) and cathode (b_peak3_ = 1.086, b_peak4_ = 0.841) peaks of Fe@FCTF Significantly higher than the b values of FCTF (0.981, 0.886 and 0.76, 0.804), indicating that its mainly capacitive‐controlled behavior, Figure S44 (Supporting Information). Quantitative analysis results show that the exfoliated Fe@FCTF electrode has faster lithium reaction kinetics and higher lithium storage capacity (at 2 mV s^−1^, the capacity contribution ratios of Fe@FCTF and FCTF are 84% and 73%, respectively), endowed with high rate capability owing to its multi‐directionally extended layered structure and widely exposed active sites, Figures 5j and S45 (Supporting Information). As shown in Figures 5k,l, and S46 (Supporting Information), using the GITT titration test analysis, the Fe@FCTF electrode exhibited a higher Li^+^ diffusion coefficient of 10^−17^‐10^−18^ cm^2^ s^−1^, while that of the FCTF electrode was only 10^−18^‐10^−20^ cm^2^ s^−1^, which can be attributed to its multi‐directionally extended layered structure, spacious interior space, and widely exposed active sites. For a better comparison, under the same test conditions, the capacity contribution diagram of stripped non‐fluorinated CTF was obtained (at 2 mV s^−1^, the capacity contribution rates of Fe@CTF and CTF were 70% and 64%, respectively), slightly lower than Fe@FCTF and FCTF, Figure S47 (Supporting Information). This shows that the introduction of F contributes to the anchoring of Fe on the surface of the layered structure, forming more active sites and improving the ion storage performance. The electrochemical performance indices of the electrodes of several materials synthesized in this paper are in Tables S9–S11 (Supporting Information).

**Figure 6:**
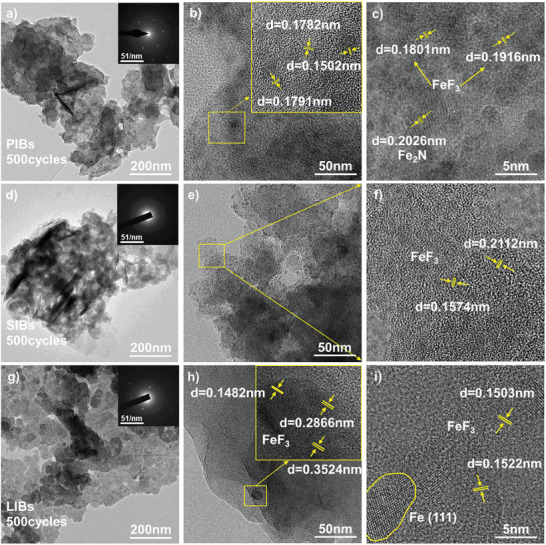
TEM images of different cells after 500 cycles of charge/discharge with Fe@FCTF as an electrode. In PIBs:a) Low‐resolution TEM image and corresponding SAED pattern. b,c) High‐resolution TEM image of different places and crystal lattice spacing. In SIBs:d) Low‐resolution TEM image and corresponding SAED pattern. e,f) High‐resolution TEM image of different places and crystal lattice spacing. In LIBs:g) Low‐resolution TEM image and corresponding SAED pattern. h,i) High‐resolution TEM image of different places and crystal lattice spacing.

To analyze the stability of the Fe@FCTF structure, the Fe@FCTF electrode materials in different cells were characterized using TEM after long cycles. TEM tests were performed on the Fe@FCTF containing conductive carbon black and the binder after 500 cycles (**Figure** **6**). Owing to the presence of the binder and the densification of the pore structure caused by SEI formation, the overall structure remained largely intact, and it is clear that the electrode had good cycling stability. The Fe@FCTF in the PIBs was calibrated, as in Figure 6a–c. Short‐range nominal carbon layers also exist in the Fe@FCTF after potassiation because of the transformation from disordered carbon to partial graphitic stacking in the short range. Remarkably, the interlayer spacing decreased to 0.1502, 0.1782, and 0.1791 nm at full potassiation, which is still larger than the size of the K^+^. Furthermore, a lattice stripe with a pitch of 0.2026 nm was observed, corresponding to the (211) atomic plane of Fe_2_N, whereas lattice fringes with spacings of 0.1801 nm and 0.1916 nm were observed, corresponding to the atomic planes of FeF_3_. The Fe@FCTF prepared after 500 cycles still exhibits a multilayer sheet petal‐like structure, as “defect adsorption” is the main sodium storage mechanism without interlayer structural changes, Figure 6d–f. In LIBs, the morphology of the Fe@FCTF composites was similar to that of the original material after 500 cycles, indicating that the conversion reaction was highly reversible, Figure 6j. Based on the TEM results, the conversion reaction of FeF_3_ can be written as:

(4) $$\mathrm{Fe}F_{3}+3Li^{+}+3e^{-}↔3\mathrm{LiF}+\mathrm{Fe}$$

Fe with a size of 0.1532 nm and different orientations were observed inside the particle after the 500th charge (Figure 6h,i). This is possibly a result of electrochemically induced grain refinement: during discharge, large FeF_3_ particles are refined into smaller Fe domains; during charging, these small Fe domains are converted back to FeF_3_, leading to the formation of smaller FeF_3_ grains.^[^ ^104^, ^105^ ^]^ The long cycling stability of the Fe@FCTF electrode is mainly attributed to the presence of functional groups on the surface of the carbon layer, which allows for the rapid adsorption and detachment of K^+^/Na^+^/Li^+^, and the microporous structure provided by the precursor CTF, which can buffer the volume expansion caused by the embedding and de‐embedding processes of K^+^/Na^+^/Li^+^.^[^ ^106^, ^107^ ^]^ At the same time, the presence of Fe improves electrical conductivity, which is conducive to rapid charge transfer, resulting in good multiplicity performance under high currents.

**Figure 7:**
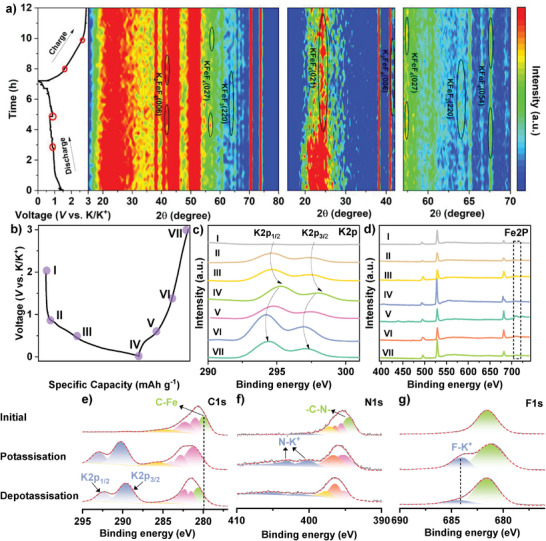
In PIBs: a) In situ XRD contour‐filled profiles and their corresponding charge/discharge curves. b) Representative charge/discharge curve of Fe@FCTF at a current density of 100 mA g^−1^. I indicate an open circuit state. II, III, and IV indicate that the initial state is discharged to 0.9, 0.5, and 0.01 V (versus K/K^+^), while V, VI, VII, and VIII indicate that the initial state is charged to 0.6, 1.2, and 3.0 V (versus K/K^+^). Ex situ XPS analysis of c) K2p, d) Fe2p, e) C1s, f) N1s, and g) F1s regions.

To better understand the electrochemical storage mechanism of Fe@FCTFs in PIBs, SIBs, and LIBs, we characterized the Fe@FCTF electrodes at different potentials using in situ XRD and ex situ XPS. **Figure** **7**a shows the first charge‐discharge curve of Fe@FCTF in a potassium‐ion battery on the left and the in situ XRD contour plot of the Fe@FCTF electrode on the right. The peak at 38.7° with a constant intensity was derived from the Be window. The results show that potassium storage has stepwise features. During the discharge process, the intensities of the peaks at 23°, corresponding to the (110) crystal planes of carbon, faded gradually until they disappeared at 0.5 V, indicating that the carbon skeleton had reacted. Reflections at 24°,42°,57°, 63°, and 67° were always observed during the charging process, which can be indexed to the K‐inserted phases of the general formula KFeFx. These results indicate that potassium storage in the Fe@FCTF was mainly due to surface adsorption and, to a lesser extent, intercalation reactions. XPS was used to investigate changes in the elemental composition and valence states of the Fe@FCTF electrode materials during the electrochemical reaction. Figure 7b shows a charge/discharge curve of the Fe@FCTF electrode at a current density of 0.1 A g^−1^. As shown in Figure 7c, the K2p peaks illustrate a negative shift during discharging and recover during followed charging, demonstrating the “rocking‐chair” potassiation/depotassiation process. A high‐resolution Fe2p XPS spectrum is shown in Figure 7d. In the XPS spectrum of the discharge/charge state, except for the Fe^2+^ and Fe^3+^ peaks, distinct Fe^0^‐related peaks appeared at the binding energy position (721.1 eV). In the charged state (3.0 V), the peak corresponding to Fe^0^ disappeared. The above variation indicates that the Fe in the Fe@FCTF plays a role in the electrochemical energy storage process.^[^ ^108^ ^]^ As can be seen in Figure 7e, the initial C1s spectrum can be deconvoluted into five peaks corresponding to C‐F (290.4 eV), C═N (286.7 eV), C‐N (285.6 eV), C‐C (284.8 eV), and C‐Fe (283.2 eV). After potassiation, two strong new peaks appear at 291.8 eV and 294.4 eV, which can be assigned to the interaction between K^+^ and Fe@FCTF. Additionally, after potassiation, the C‐N peak intensity increased, and the C═N peak intensity decreased, suggesting the transformation of the latter C═N bonds to C‐N‐K groups through combination with K^+^. During the depotassiation process, the two peaks due to C‐K^+^ were substantially weakened, while those attributed to C═N were strengthened, suggesting K^+^ de‐intercalation from the Fe@FCTF framework during this procedure. In the high‐resolution N1s (Figure 7f), the N1s peak was fitted to five peaks. The C‐N (398.5 eV) and C‐N (399.68 eV) peaks remained stable throughout the charging and discharging processes. The new peak at 403.2 eV observed upon complete potassiation can be attributed to the interaction of K^+^ with N in the triazine ring. The Fe‐N (397.4 eV) signal disappeared after discharging and reappeared until charging to 3 V, demonstrating that the Fe‐N bond was broken during discharging and reestablished during charging. In the F1s region (Figure 7g), the spectra are dominated by peaks that can mainly be assigned to KF resulting from KFSi decomposition.

The in situ XRD contour plots of the Fe@FCTF electrodes in SIBs and their corresponding charge/discharge curves are shown in Figure S48 (Supporting Information). As the discharge proceeds, the peak at ≈23° gradually disappears. Meanwhile, new diffraction peaks appeared at 33.5°, 46°, 54°, and 67°, which could be related to the metal sodium, demonstrating the appearance of sodium‐intercalated products in the electrode. The emerging diffraction peaks are attributed to NaFeOx. Figure S49a (Supporting Information) shows the charge/discharge curves of Fe@FCTF electrodes applied to SIBs at a current density of 0.1 A g^−1^. As shown in Figure S49b (Supporting Information), the Na1s peak was similarly negatively shifted during discharging and recovered during subsequent charging, suggesting a rocking chair potentiation/depotentiation process.^[^ ^67^, ^109^ ^]^ The XPS spectrum is shown in Figure S49c (Supporting Information). In the XPS spectra of the discharged/charged states, a distinct peak associated with iron oxide appeared at a higher binding energy (710.1 eV) than that of the initial state. This is consistent with the phenomenon observed in the in situ XRD results. This suggests that the Fe in Fe@FCTF plays a role in the sodium storage process. As shown in Figure S49d (Supporting Information), the initial C1s spectrum can also be deconvoluted into five peaks. After potassiation, two strong new peaks appear at 292.8 eV and 295.4 eV, which can be assigned to the interaction between Na^+^ and Fe@FCTF. In addition, the disappearance of the C‐Fe peak after sodiation suggests that the C‐Fe bond was broken, contributing to Na^+^ storage. As the denaturation process proceeded, the two peaks of C‐Na^+^ production diminished dramatically, and C‐Fe reappeared, suggesting that sodium may first be separated from the organic ligand during this process, after which the C‐Fe bond begins to reorganize. In the high‐resolution N1s (Figure S49e, Supporting Information), the N1s peak was fitted to three peaks. The C‐N (398.5 eV) and C‐N (399.68 eV) peaks remained stable throughout the charging and discharging processes. The Fe‐N (397.4 eV) signal disappeared after discharging and reappeared until charging to 2.5 V, demonstrating that the Fe‐N bond was broken during discharging and reestablished during charging. In the F1s region (Figure S49f, Supporting Information), the spectra were dominated by peaks that could mainly be assigned to NaF, resulting from NaPF_6_ decomposition.

The in situ XRD contour plots of the Fe@FCTF electrodes in LIBs and their corresponding charge/discharge curves are shown in Figure S50 (Supporting Information). The intensity of the peak at 23°, corresponding to the (110) crystal plane of the carbon, is also found to diminish with the lithiation process in the LIB until it disappears at 1.5 V, still suggesting that it is the carbon skeleton that is reacting. Unlike potassium ^−1^sodium, the characteristic C peak at 23° reappears when charging to 1.2 V. This indicates that the embedding and de‐embedding of Li in the carbon skeleton during the reaction was fully reversible. In addition, the characteristic peak at 21°, corresponding to FeF_3_ (001), changed from strong to weak during the physicochemical process. Characteristic peaks were observed at 66° and 43°, attributable to the (200) and (220) crystal planes of LiF, respectively. A new diffraction peak was observed at 82°, which was attributed to the (111) crystal face of Fe. The resulting transformation corresponds to:

(5) $$\mathrm{Fe}F_{3}+\mathrm{Li}+e^{-}↔\mathrm{LiFe}F_{3}$$

(6) $$\mathrm{LiFe}F_{3}+2\mathrm{Li}+2e^{-}↔\mathrm{LiF}+Fe^{0}$$

These results indicate that the lithium storage in Fe@FCTF is mainly due to a combination of intercalation reactions and surface adsorption, which is consistent with the results of the CV and non‐in situ TEM tests.^[^ ^110^ ^]^ Figure S51a (Supporting Information) shows the charge/discharge curves of the Fe@FCTF electrodes in a LIB at a current density of 0.1 A g^−1^. As shown in Figure S51b (Supporting Information), the Li1s peak was similarly negatively shifted during discharging and recovered during subsequent charging, suggesting a rocking chair potentiation/depotentiation process. In addition, the discharge to 0.01 V showed a new peak at 63 eV, which can be attributed to the formation of LiFe. In the XPS spectra of Fe (Figure S51c, Supporting Information), the ratio of Fe^3+^/Fe^2+^ gradually decreases with the lithiation process up to 0.01 V, where Fe^0^ appears at the lower binding energy. The ratio of Fe^3+^/Fe^2+^ gradually recovered during the delithiation process. This suggests that the change in the iron content in Fe@FCTF is reversible and plays a vital role in the lithium storage process. The C1s spectrum (Figure S51d, Supporting Information) had four fitted peaks located at 283.5, 284.8, 286.2, and 289.9 eV, which can be attributed to the C‐Fe group, C‐C group, C═N group, and strong C‐F ionic bonds, respectively. During the discharge process (lithium insertion), from the initial voltage to 1.5 V and then to 0.01 V, the peak intensity of the C═N functional group gradually weakened, while that of the C‐N functional group gradually increased. This can be attributed to the gradual conversion of the C═N groups into strong C‐N‐Li ionic bonds during lithium insertion. The peak intensity changes for the C═N and C‐N groups were not significant. Notably, the sp2 hybridized C═N peak intensity decreased significantly and then increased during the gradual discharge and charge process to 3.0 V (versus Li/Li^+^), which is consistent with the in situ XRD results. As shown in Figure S51e (Supporting Information), the N‐Fe bond disappeared after complete lithiation, and an N‐Li bond appeared, which corresponded to a newly formed C‐N‐Li ionic bond. The N‐Fe bonds reappeared after charging to 3 V but were significantly weaker. The presence of LiF only after complete lithiation can be attributed to the oxidation of Li to Li^+^ by FeF_3_ (Figure S51f, Supporting Information).

**Figure 8:**
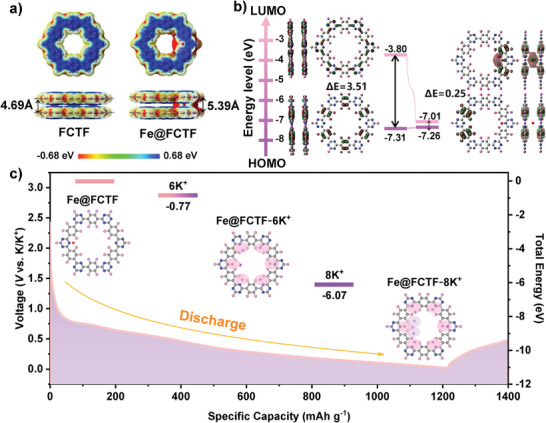
a) Electrostatic potential of Fe@FCTF and FCTF. b) LUMO energy level, HOMO energy level, and HOMO‐LUMO energy level potential difference of Fe@FCTF and FCTF. c) Proposed potassiation pathway for Fe@FCTF electrode.

To further validate the redox reaction of the potassium storage mechanism at this electrode, calculations were performed by applying first principles (DFT), discussing the adsorption sites and adsorption energies of Fe@FCTF and FCTF on K^+^, and simulating the potassiation process of the Fe@FCTF structure. A Gaussian simulation program was used to calculate the optimized Fe@FCTF and FCTF structures, as shown in Figure S52 (Supporting Information). The electrostatic potential band represents the structure's electronegativity magnitude, where red represents negative electricity and blue represents positive electricity (**Figure** **8**a). Darker colors represent greater absolute values. In the optimized Fe@FCTF and FCTF structures, the C═N group in the triazine structure shows a strong negative charge, which is a clear indication that this active site can effectively bind K^+^. Owing to the offsetting effect of the benzene ring, positive fluoride ions in the inner structures of Fe@FCTF and FCTF and the negative charge of the inner position of the C═N group are not evident. Notably, the intensity of the negative charges in the Fe@FCTF structure is closer to that of Fe. This suggests that the presence of Fe in the Fe@FCTF structure provides additional active sites for K^+^. In addition, according to the calculation results, the interlayer spacing between the modified Fe@FCTF is significantly larger than that of FCTF (4.69 Å) to be at 5.39 Å. Larger interlayer spacing facilitates electrolyte injection and ion transport.

As shown in Figure 8b, the energies of the LUMO/HOMO orbitals for Fe@FCTF were calculated to be −7.01/−7.26 eV, accompanying a narrow band gap (0.25 eV), which are lower than those for FCTF (band gap = 3.51 eV, LUMO = −3.80 eV; HOMO = −7.31 eV). This suggests that the Fe@FCTF has a greater electron affinity and a higher reduction potential. The decreased HOMO and LUMO values, along with the reduced band gap, are beneficial for fast ion/electron transfer and improved electrical conductivity and electrochemical performance of the Fe@FCTF material for PIBs, SIBs, and LIBs.^[^ ^72^, ^111^, ^112^ ^]^ The adsorption energies for combining 1, 6, and 8 K^+^ at different positions were also simulated and calculated. The initial state was denoted as Fe@FCTF, and the K^+^ bound to the C═N group was denoted as Fe@FCTF‐nK. The K^+^ moved reversibly between the layers in the Fe@FCTF structure (Figure S53, Supporting Information). Fe@FCTF‐6K denotes K^+^ populated with C═N groups, which populate the six active sites in the interlayer with an adsorption energy of −0.77 eV. Fe@FCTF‐8K denotes 8 K^+^, filling almost all the active sites with an adsorption energy of −6.81 eV. These two states exhibited excellent stability.^[^ ^113^ ^]^ As a result, up to eight potassium ions could theoretically interact with each Fe@FCTF repeating unit, resulting in a theoretical specific capacity of 1290 mAh g^−1^. These results demonstrate that a discharge platform at ≈0.9 V exists due to the strong coordination of the N atom in the C═N group with potassium ions, which can increase the storage capacity of the electrode through a multi‐electron redox reaction.^[^ ^114^ ^]^ Furthermore, these results are in good agreement with the Li storage mechanism shown in Figure 8c. This phenomenon was also observed in non‐in situ XPS. However, this is only an idealized potassium storage mechanism, and the actual mechanism is very complicated.

## 3 Conclusion

In summary, this study provides a simple and safe molten salt exfoliation strategy for layer‐expanding 2D materials, leading to the preparation of novel organic composites with high stabilities and rate capabilities. The multi‐directional extended layered structure broadened by effective exfoliation to expose more embedding sites endows the Fe@FCTF anode with a high K^+^ storage capability in PIBs. Characterization by AFM confirmed that the exfoliated Fe@FCTF had a spacious laminar structure, facilitating electrolyte injection and ion transport. The test results showed that the reversible specific capacity and cycle performance of Fe@FCTF were improved. At 0.1 A g^−1^, the potassium capacity after 100 cycles is 447 mAh g^−1^; at 0.5 A g^−1^, the potassium capacity is 352 mAh g^−1^ after 500 cycles. The Na and Li capacities are 357 mAh g^−1^ or 988 mAh g^−1^, respectively, after 500 cycles. In situ XRD, ex situ XPS, and DFT calculations were performed to reveal the K storage mechanism of the Fe@FCTF electrode. The high reversible capacity of this electrode was attributed to its C═N functional and iron coordination groups. These results will facilitate the design and preparation of layered covalent organic frameworks with excellent electrochemical properties and potential applications as anode materials for alkali metal‐ion batteries.

## 4 Experimental Section

### Materials

All chemicals and reagents were obtained from Dodo Chemical Reagents and the online chemical platform of Tianjin Normal University and were used without further purification. Zinc chloride (ZnCl_2_, 98%) and anhydrous ferric trichloride (FeCl_3_, 97%) were obtained from the Yuanli Chemical Company and stored in a glovebox to prevent moisture and oxidation. Acetylene black AB (>99%), 2,3,5,6‐tetrafluoro‐1,4‐phenylene terephthalonitrile (C_8_F_4_N_2_, 97%), and 1,4‐phenylene terephthalonitrile (C_8_H_4_N_2_, 98%) were purchased from Maclean Chemical. Lithium hexafluorophosphate (LiPF_6_), sodium hexafluorophosphate (NaPF_6_), and potassium bis(fluorosulfonyl) (KFSI) with certain proportional concentrations were purchased from Dodo Reagent Networks. Fe@FCTFs, FCTF, Fe@CTFs, and CTF were synthesized using literature methods.

### Preparation of FCTF and CTF by Self‐Polymerization

In a typical ionothermal synthesis experiment, zinc chloride in a molten state acting as a catalyst and tetrafluoroterephthalonitrile (1 mol) were mixed in a ratio of 1:1 and transferred to a 1.5 × 1.5 × 20 cm^3^ ampoule under an argon atmosphere. The ampoule was then evacuated, sealed well, transferred to a muffle furnace, and heated at 350 °C for 40 h to obtain a black fluffy product. The FCTF in ≈80% yield was obtained by washing with deionized water and anhydrous ethanol (>99%) in several draws to remove residual zinc chloride, followed by drying in a vacuum oven at 80 °C for 15 h. CTF can be achieved similarly, only using terephthalonitrile instead of tetrafluoroterephthalonitrile.

### Preparation of Fe@FCTF and Fe@CTF by Molten Salt Stripping Method

The FCTF was thoroughly mixed with anhydrous ferric chloride in a ratio of 1:4 and transferred to a 1.5 × 1.5 × 20 cm^3^ ampoule under an argon atmosphere. The ampoule was then evacuated, sealed, transferred to a muffle furnace, and heated at 360 °C for 72 h to obtain a greenish‐brown product. The stripped FCTF product (Fe@FCTF) was obtained by washing with deionized water and anhydrous ethanol (>99%) several times via filtration to remove residual ferric and ferrous chlorides, followed by drying in a vacuum oven at 80 °C for 15 h. Exfoliated Fe@CTF was obtained using a method similar to that of the non‐fluorinated CTF precursor. (One thing to keep in mind is that when we vacuum the ampoule, the ampoule is connected to the tube sealer by a short transfer in air, a process that may result in the oxidation of some of the anhydrous ferric trichloride, generating iron oxides inside the ampoule.)

### Material Characterization

The microstructures of the samples were observed using scanning electron microscopy (SEM, S‐4700, Hitachi, Japan) and high‐resolution transmission electron microscope (HR‐TEM, JEM‐2000, EX, JEOL, Japan). X‐ray diffraction (XRD, Rigaku Ultima IV, Japan) was performed at a voltage of 40 kV and a current of 40 mA. The crystalline and physical phases of the materials were analyzed using monochromatic CuKα radiation (λ = 1.5418 nm) passed at a scanning speed of 5° min^−1^ under the conditions. Raman spectroscopy (Raman, HORIBA LabRAM HR Evolution) was performed using a 532 nm semiconductor laser as the excitation source in the 50–4000 cm^−1^ wavenumber range. XPS analyses were conducted with a spectrometer using focused monochromatized Al Kα radiation (Thermo Fisher, ESCALAB 250Xi, USA). The full spectrum of the pass energy was 100 eV, the elemental high‐resolution spectrum was 30 eV, and charge calibration was performed at C1s = 284.8 eV. The energy of argon ion etching was 2 keV, and the beam current was 2 uA with 3 keV. Fourier transform infrared (FTIR) spectra were obtained using a Bruker spectrometer with a KBr pellet in the frequency range of 500–4000 cm^−1^. Atomic force microscopy (AFM) was performed using a Brooke (United States) Multi‐Mode 8 instrument to measure the height profile of the sample. Nitrogen adsorption/desorption curves were obtained by Brunauer‐Emmett‐Teller (BET) measurements using a Micromeritics ASAP 2010 surface area analyzer. Thermogravimetry (TG) measurements were performed using a Swiss Mettler TGA/DSC 3+ instrument. Nitrogen was used as the carrier gas. The samples were programmably heated from 30 °C to 1000 °C at a heating rate of 5 °C min^−1^. The mass‐loss rate was obtained based on the first‐order derivative of the time trace of the mass loss. Electrical conductivity was measured using an RTS‐8 four‐probe tester.

### In Situ XRD Measurement

In situ XRD patterns of Fe@FCTF electrode were collected on the Bruker D8 Advance Diffractometer with a scanning rate of 5° min^−1^. A current density of 50 mA g^−1^ was selected for discharging and charging processes. The electrodes were prepared by mixing the active material with a conductive agent and PAA at a weight ratio of 6:3:1 using deionized water as the solvent. The doughy mixture was extruded onto a copper grid to form the electrodes and dried in a vacuum oven at 60 °C for 12 h before being assembled into cells in a glove box.

### Computational Details

All the calculations were performed in the Gaussian16 package at the B3LYP/DEF2‐TZVP theoretical level. The geometries of all structures were fully optimized, frequency calculations were performed at the same theoretical level as optimization to confirm that no negative frequency was observed in any of the optimized structures, and the zero‐point energy (ZPE) correction was calculated for the thermal energy value. The electrostatic potential (ESP) was analyzed using optimized structures to probe possible K^+^ binding sites. The LUMO‐HOMO energy theoretical spectra were computed via Gaussian16 software.

### Electrochemical Measurements

A coin cell (CR2032) was assembled in a glove box under an argon atmosphere, and its electrochemical properties were tested after standing for 24 h. To prepare the electrodes, 60 wt.% of the active substances (Fe@FCTF, FCTF, Fe@CTF, or CTF), 30 wt.% acetylene black, and 10 wt.% PAA were mixed in an aqueous solution to form a homogeneous slurry. The copper foil‐loaded electrode slurry (≈1.5 mg cm^−2^) was vacuum dried at 80 °C for 24 h with 1 molar concentration of LiPF_6_ in EC: DEC = 1:1 Vol% with 30% FEC, NaPF_6_ in DIGLYME = 100 Vol% or KFSi in EC:DMC: EMC = 1:1:1 Vol% for the electrolyte, while lithium, sodium, and potassium pure metals were extruded into discs of ≈12 mm diameter and used as counter electrodes for LIBs, SIBs, and PIBs, respectively. Polyethylene membranes were used as separators in LIBs, polyethylene membranes were used as separators in SIBs, and glass fibers (Whatman) were chosen as separators in PIBs. Cyclic voltammetry (CV) and electrochemical impedance spectroscopy (EIS) data were acquired using an electrochemical workstation (Princeton). Cyclic voltammetry (CV) tests were performed at scan rates of 0.1, 0.2, 0.4, 0.6, 0.8, 1, and 2 mV s^−1^, with measured voltages ranging from 0.0–3.0 V (LIBs and PIBs) or 0.0‐2.5 V (SIBs). EIS was performed in the 0.01 to 100 000 Hz frequency range, and an amplitude voltage of 5 mV was tested. Throughout this study, the capacities were calculated from the total weights of Fe@FCTF, FCTF, Fe@CTF, and CTF. The constant current intermittent titration technique (GITT) was evaluated using the LAND multi‐channel battery test system in the voltage range of 0.0‐3.0 V (LIBs and PIBs) or 0.0‐2.5 V (SIBs), and constant current charge/discharge tests, rate capability tests, and cycling performance tests were completed.

## Conflict of Interest

The authors declare no conflict of interest.

## Supporting information

Supporting Information

## Data Availability

The data that support the findings of this study are available from the corresponding author upon reasonable request.
