# Supplementary material for: Construction of High‐Performance Anode of Potassium‐Ion Batteries by Stripping Covalent Triazine Frameworks with Molten Salt
Source: Adv Sci (Weinh). 2024 Jun 26;11(32):2401804. doi: 10.1002/advs.202401804 (PMC11348138; doi:10.1002/advs.202401804)
Supplement: Supplementary file 1 — Supporting Information [file ADVS-11-2401804-s001.docx]

**Supporting Information**

**Construction of high-performance anode of potassium-ion batteries by stripping covalent triazine frameworks with molten salt**

Jingyi Zhang^1^, Xuwang Fu^1^, Jiacheng Qiu^1^, Chao Wang^1^, Li Wang^1^, Jianmin Feng^1*^, Lei Dong^1^, Conglai Long^1^, Xiaowei Wang^2*^ and Dejun Li^1*^

^1^College of Physics and Materials Science, Tianjin Normal University, Tianjin 300387, China.

^2^ National Engineering Laboratory for High Efficiency Recovery of Refractory Nonferrous Metals, School of Metallurgy and Environment, Central South University, Changsha 410083, PR China.


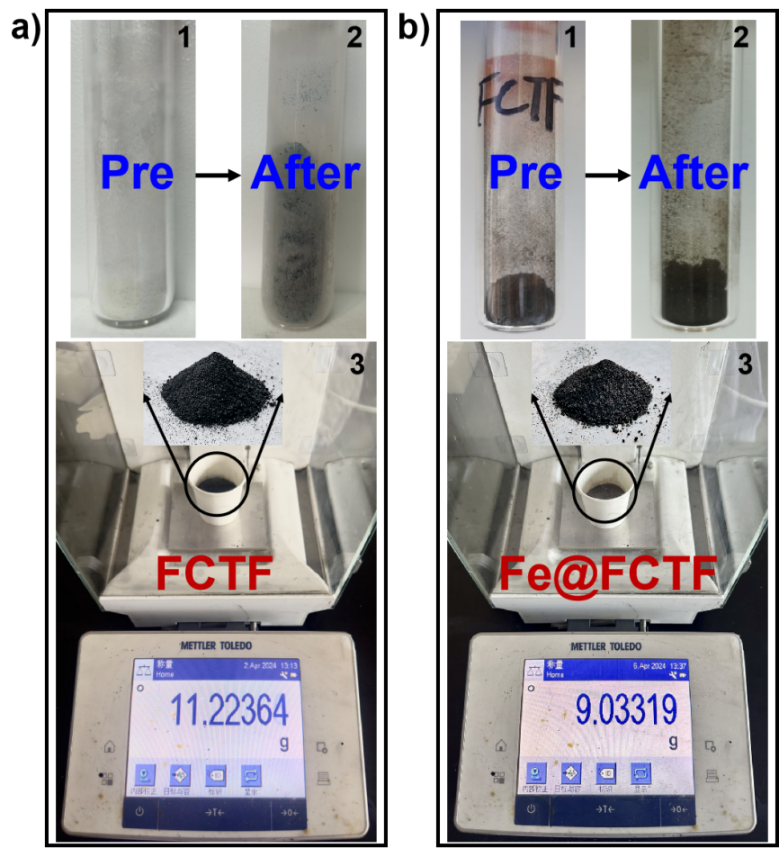


Figure S1: Preparation process and yield weighing of Fe@FCTF and FCTF samples.


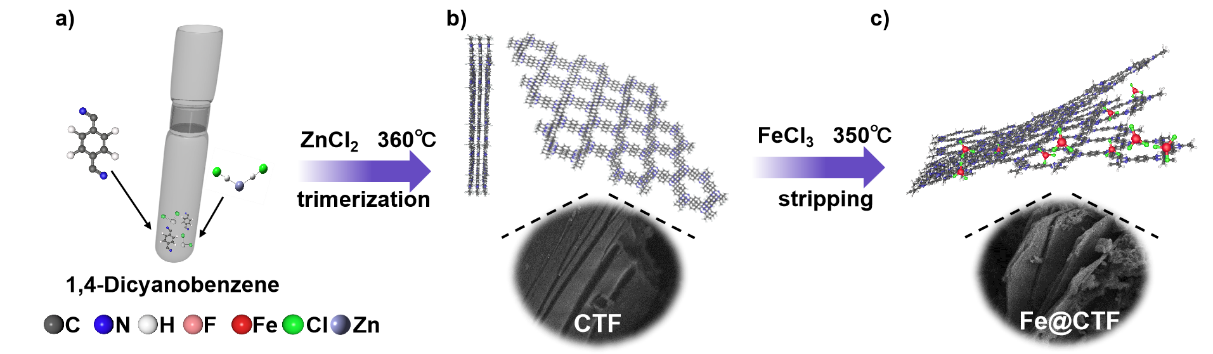


Figure S2: The ion thermal synthesis path developed in this work is used to construct CTF and Fe@CTF.


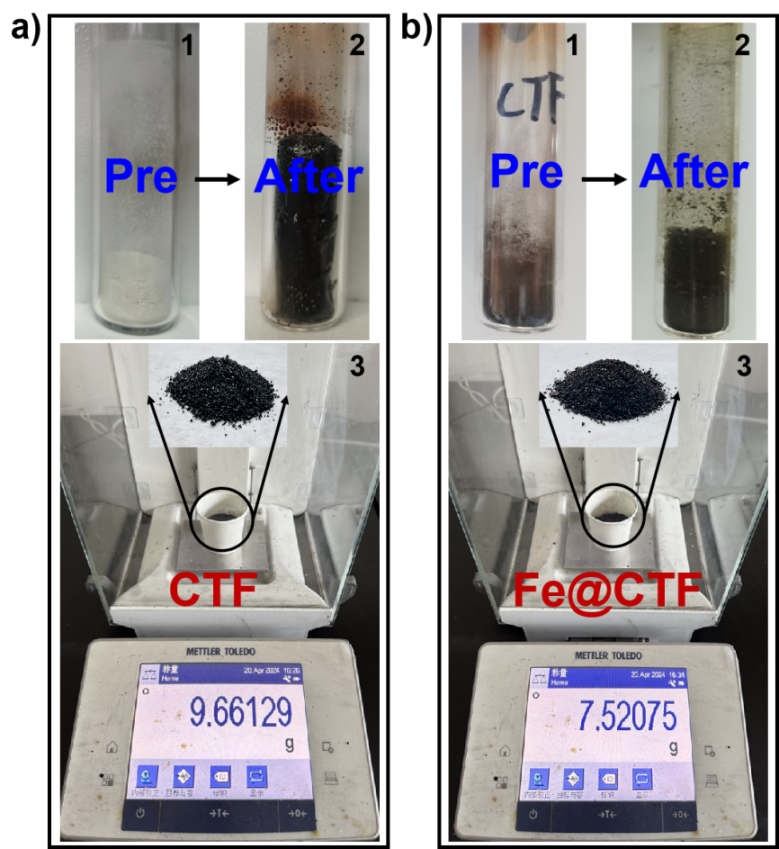


Figure S3: Preparation process and yield weighing of Fe@CTF and CTF samples.


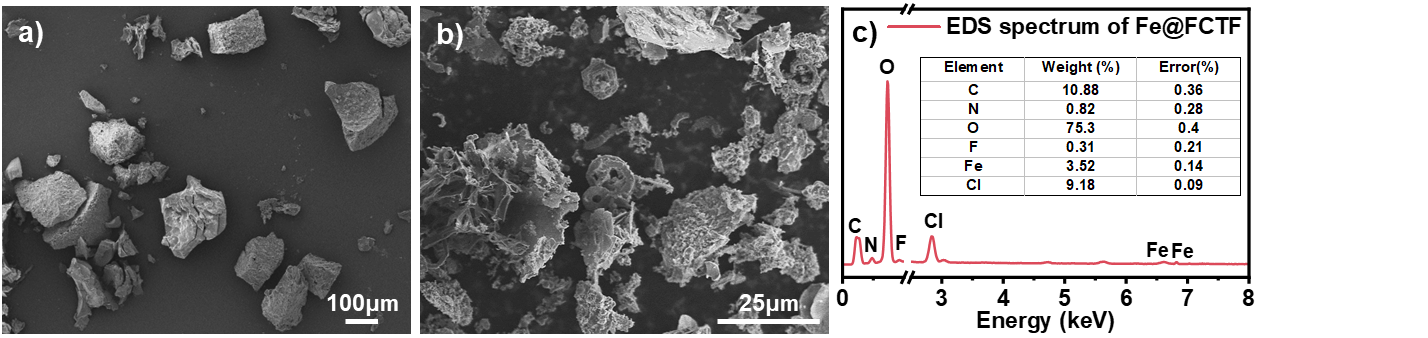


Figure S4: a, b) SEM images of Fe@FCTF at different magnifications. c) The corresponding EDS spectrum of the area in Figure 2b.


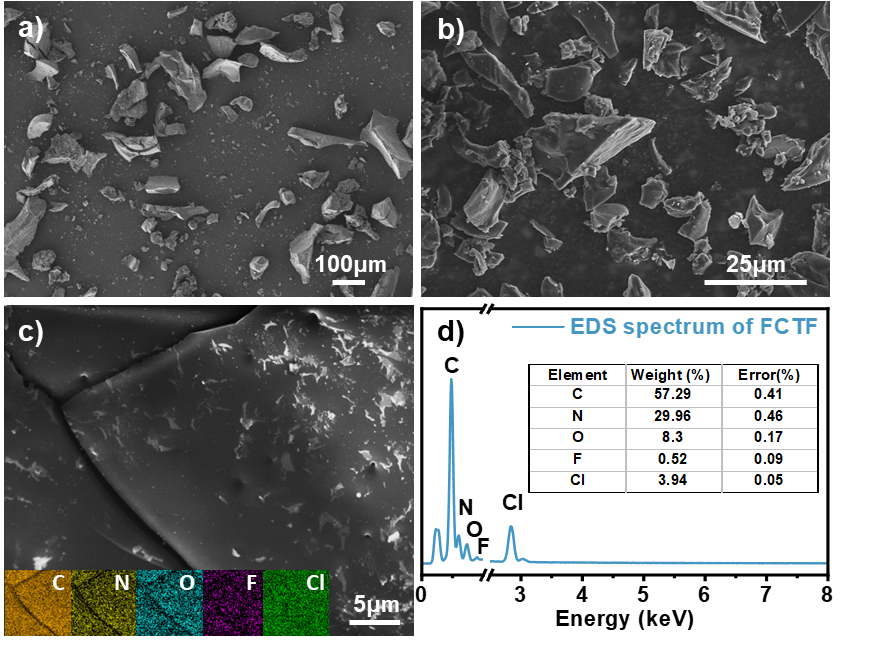


Figure S5: a, b) SEM images of Fe@FCTF at different magnifications. c) The corresponding EDS spectrum of the area in Figure 2b.


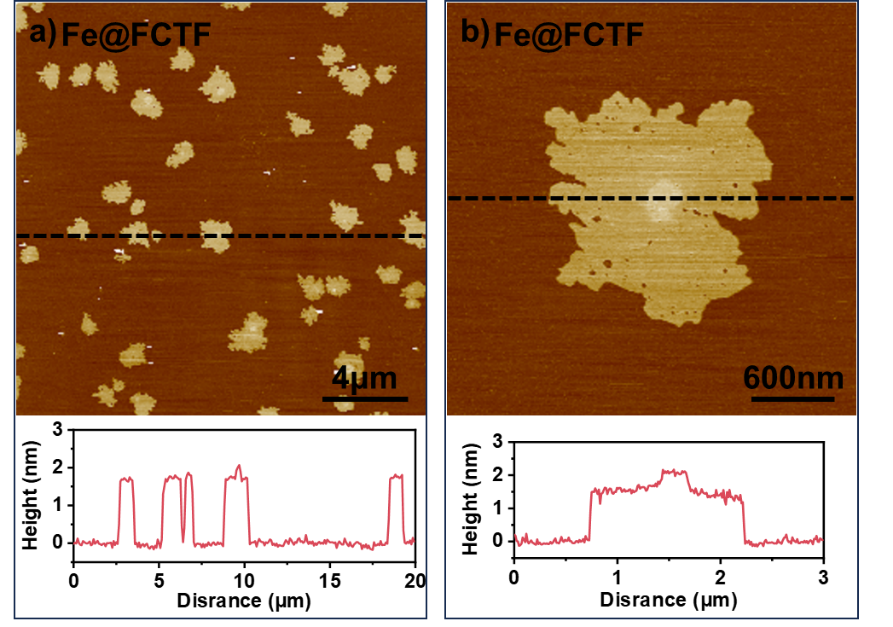


Figure S6: AFM measurements of Fe@FCTF and the corresponding height profiles for the selective regio.


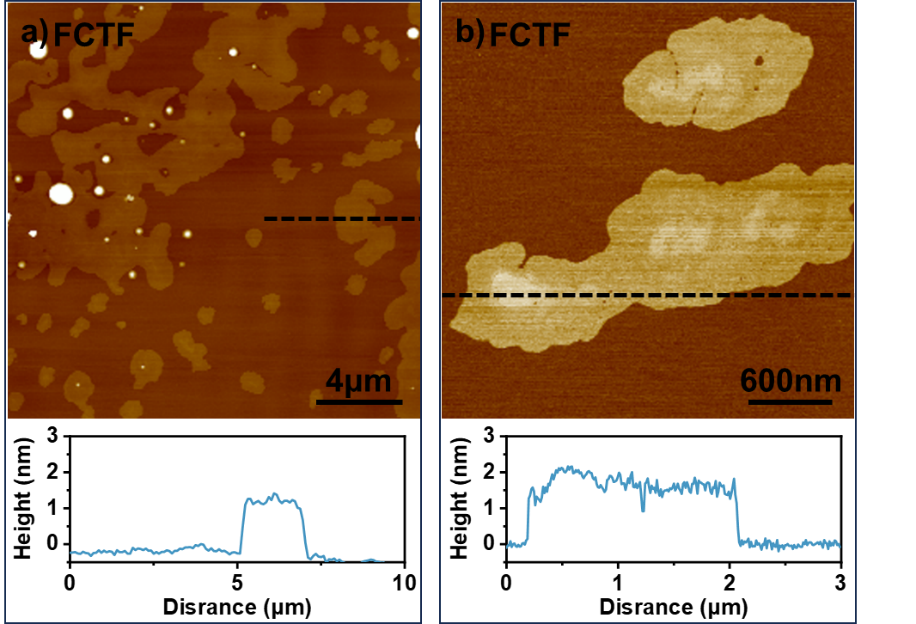


Figure S7: AFM measurements of FCTF and the corresponding height profiles for the selective regio.


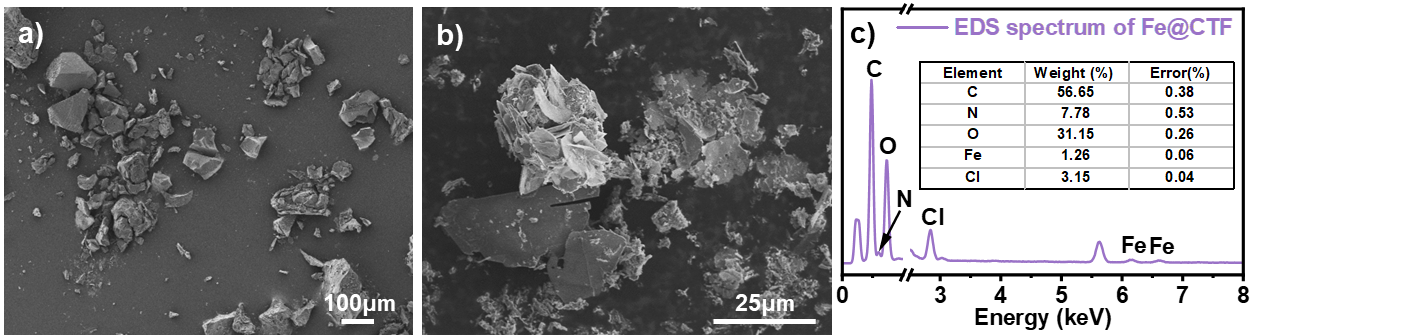


Figure S8: a, b) SEM images of Fe@CTF at different magnifications. c) The corresponding EDS spectrum of the area in Figure 2k.


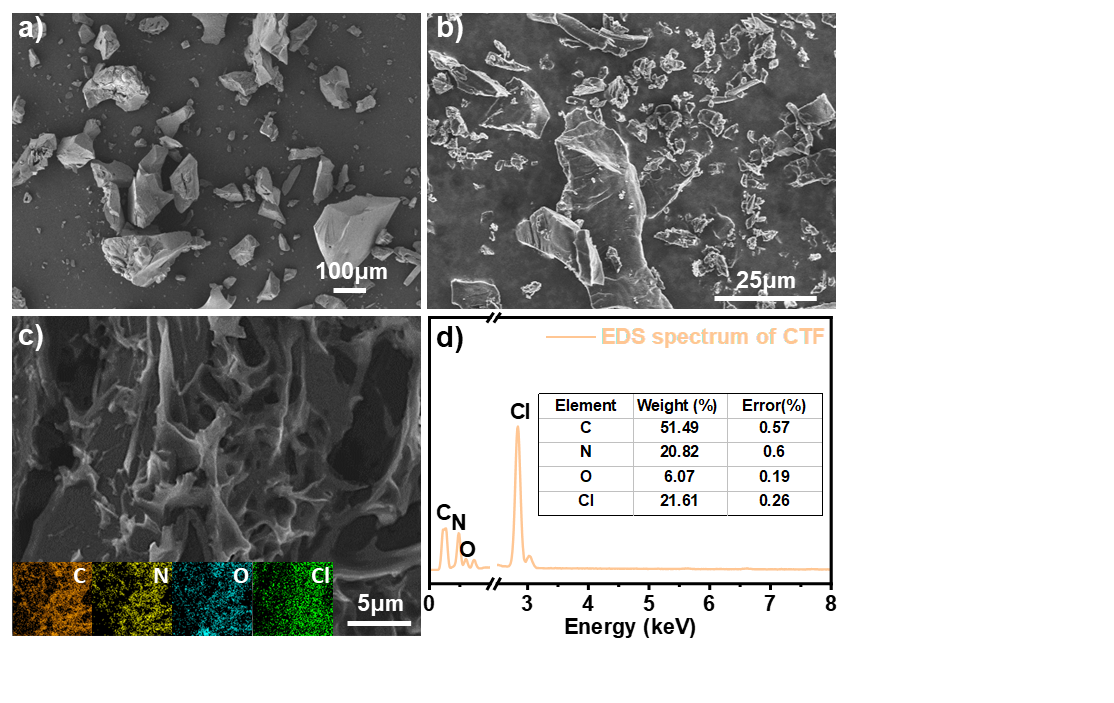


Figure S9: a, b) SEM images of Fe@FCTF at different magnifications. c) The corresponding EDS spectrum of the area in Figure 2b.


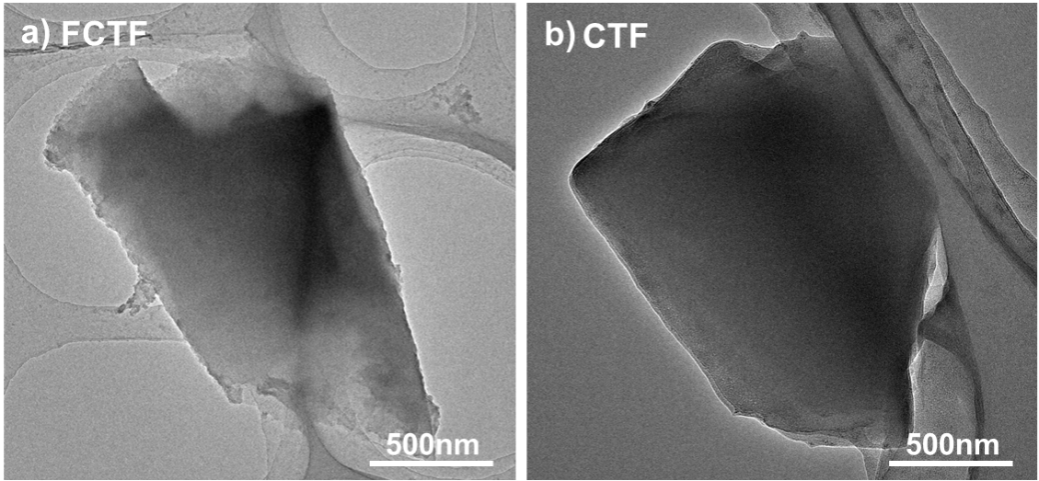


Figure S10: a) Low-resolution TEM image of FCTF. b) Low-resolution TEM image of CTF.


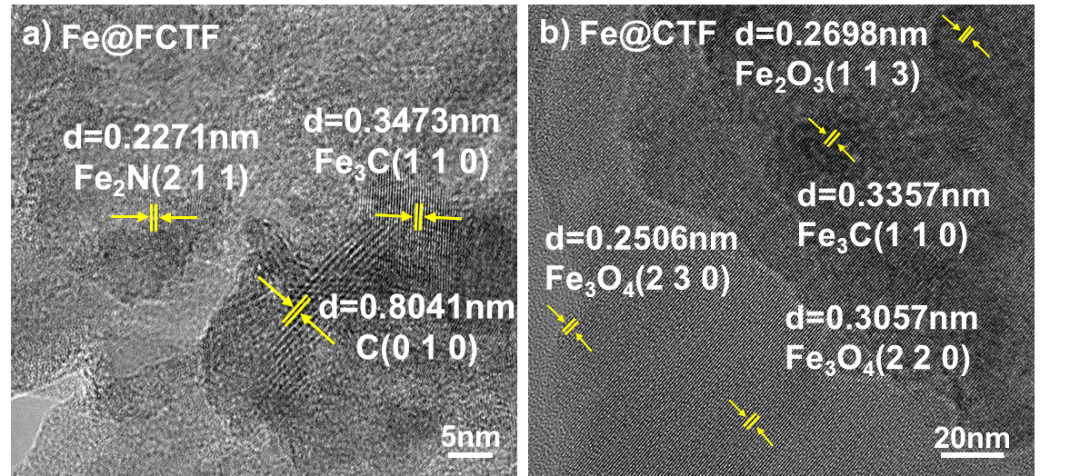


Figure S11: a) High-resolution TEM image of Fe@FCTF. b) High-resolution TEM image of Fe@CTF.


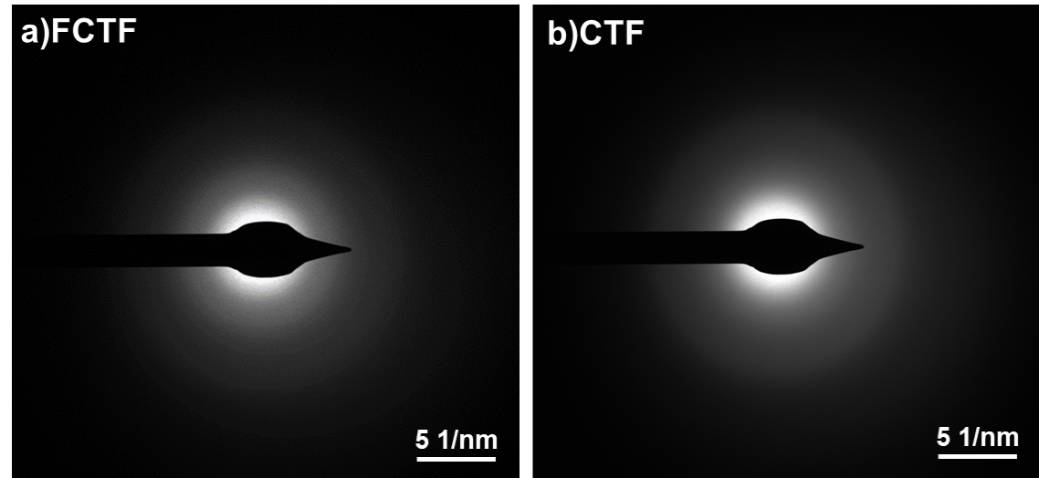


Figure S12: a) SEAD pattern of FCTF. b) SEAD pattern of CTF.


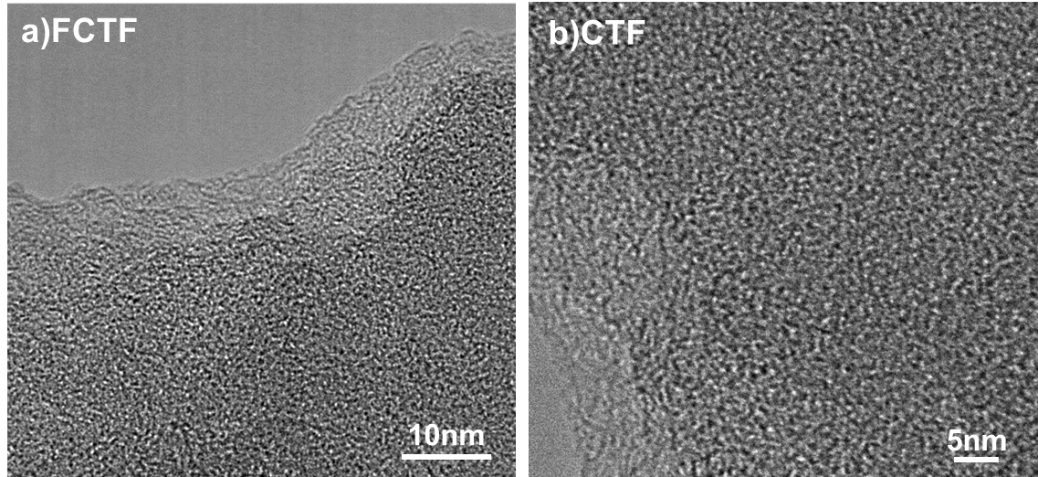


Figure S13: a) High-resolution TEM image of FCTF. b) High-resolution TEM image of CTF.


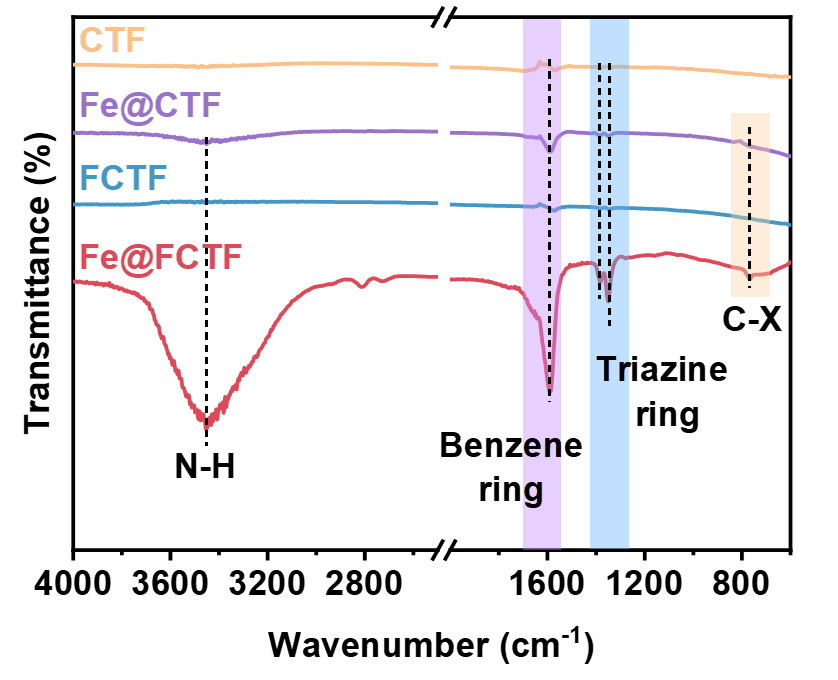


Figure S14: a) High-resolution TEM image of FCTF. b) High-resolution TEM image of CTF.


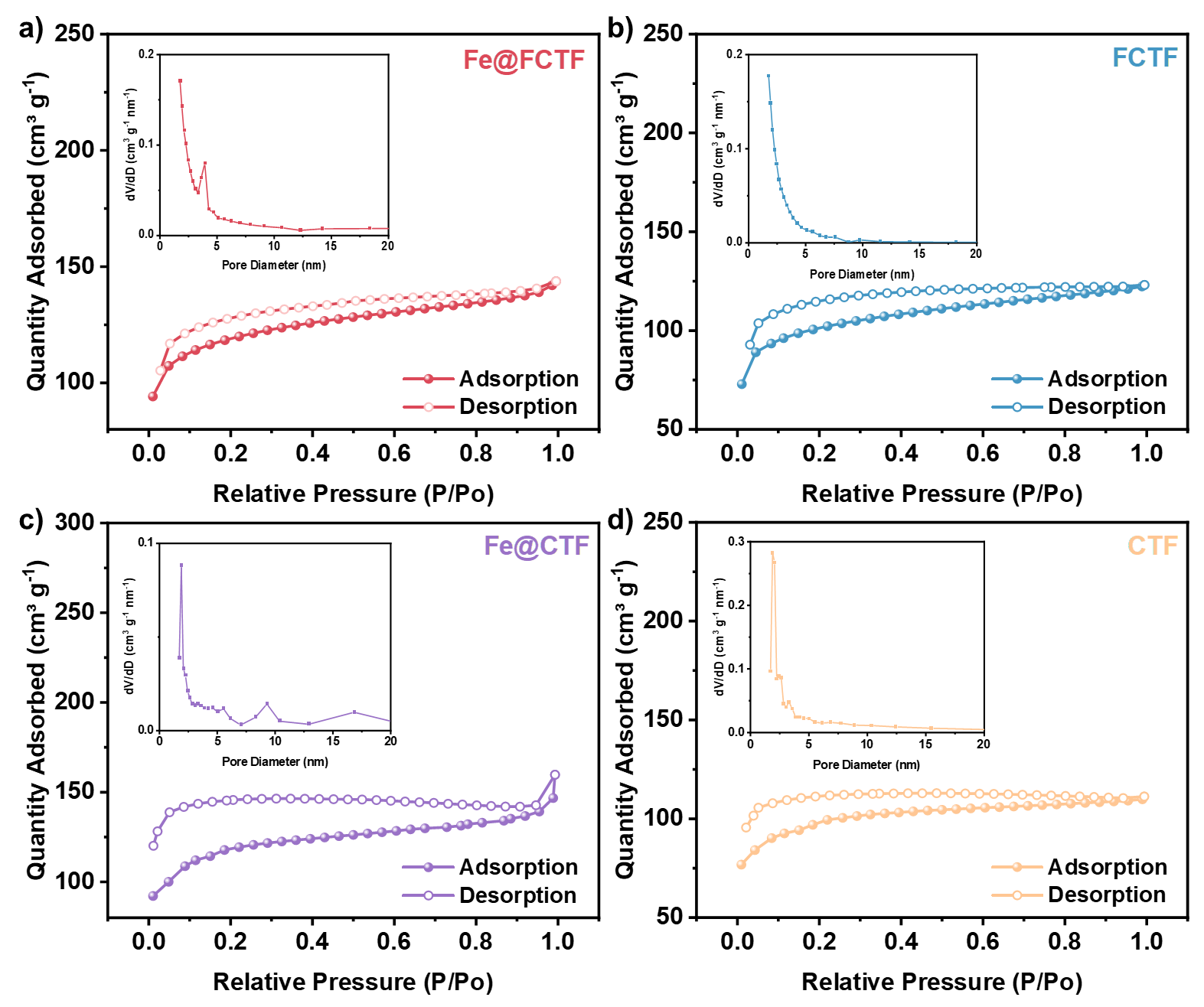


Figure S15: a) High-resolution TEM image of FCTF. b) High-resolution TEM image of CTF.

Figure S16: a) High-resolution TEM image of FCTF. b) High-resolution TEM image of CTF.


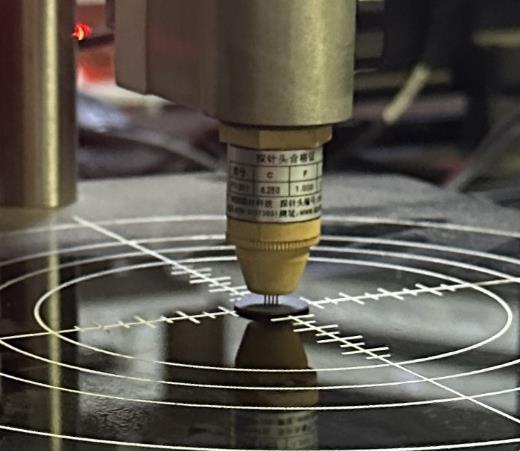


Figure S17: Four-probe method for testing the conductivity of materials.


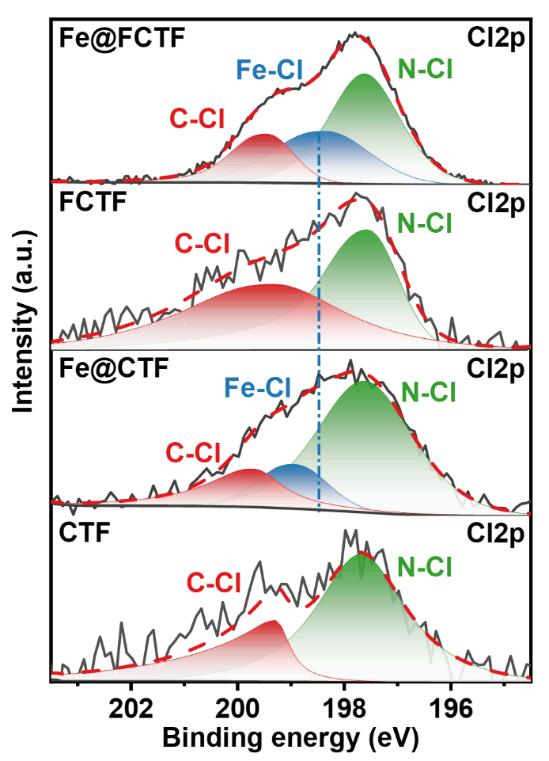


Figure S18: XPS spectra of Fe@FCTF, FCTF, Fe@CTF, and CTF: Cl 2p.


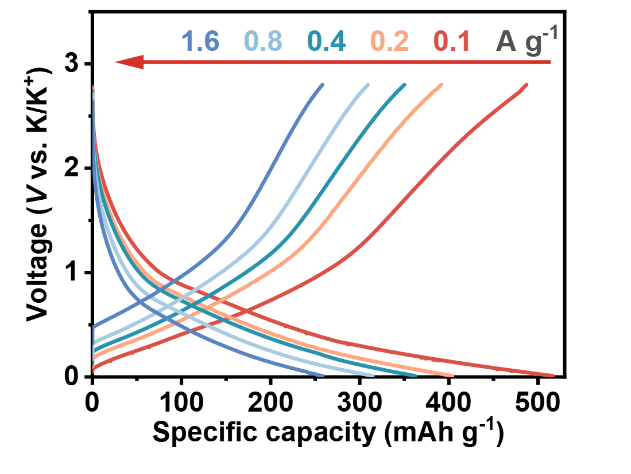


Figure S19: PIBs: Charge/discharge curves under different current densities of Fe@FCTF (corresponding to rate performance in the 5th, 15th, 25th, 35th, and 45th cycle in Figure 4c).


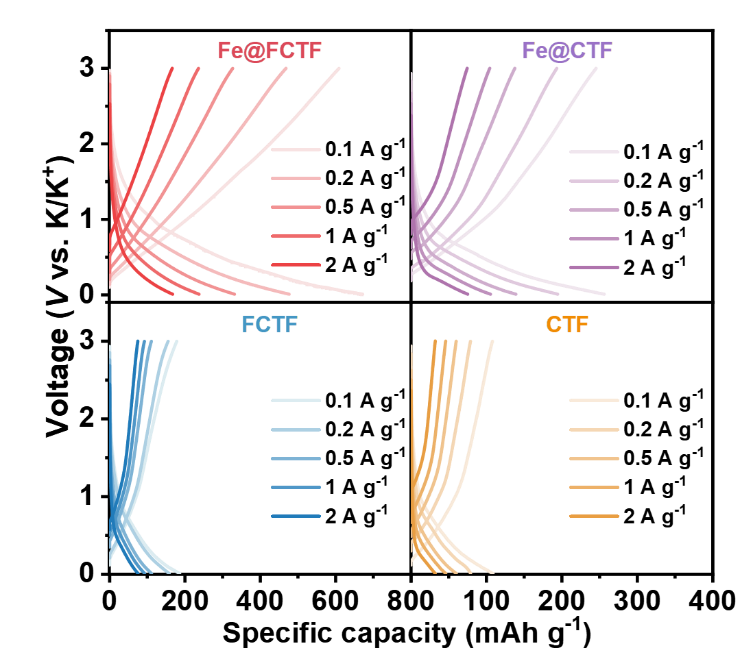


Figure S20: PIBs: Charge/discharge curves under different current densities of the Fe@FCTF and other CTFs.


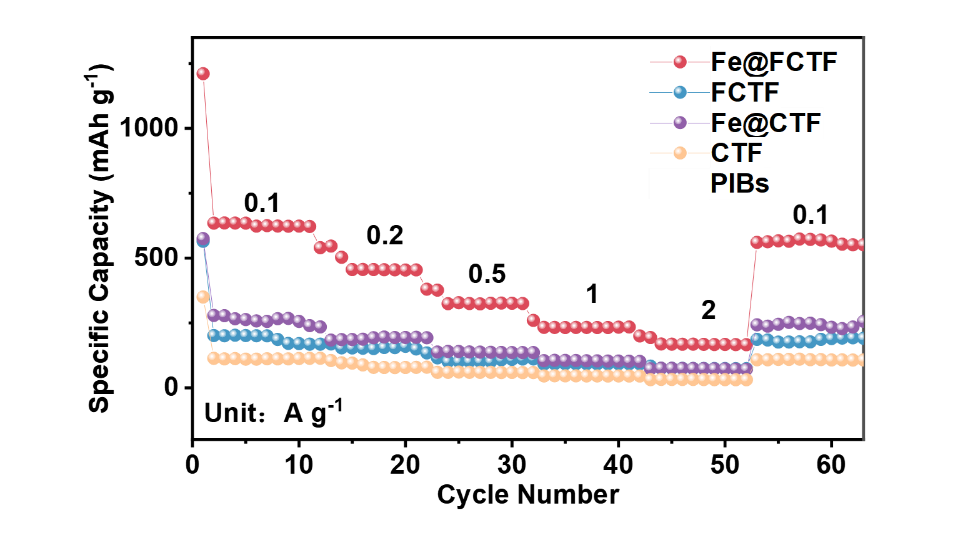


Figure S21: PIBs: The rate capability of Fe@FCTF and other CTFs from 0.1-2 A g^-1^.


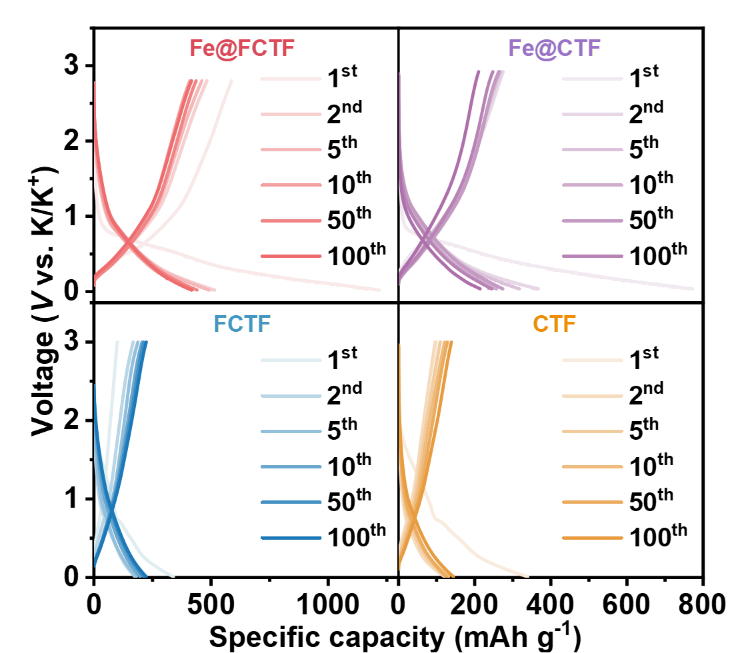


Figure S22: PIBs: Charge/discharge curves of the Fe@FCTF and other CTFs electrodes at 0.1 A g^-1^ for different cycles are in Figure 4d.


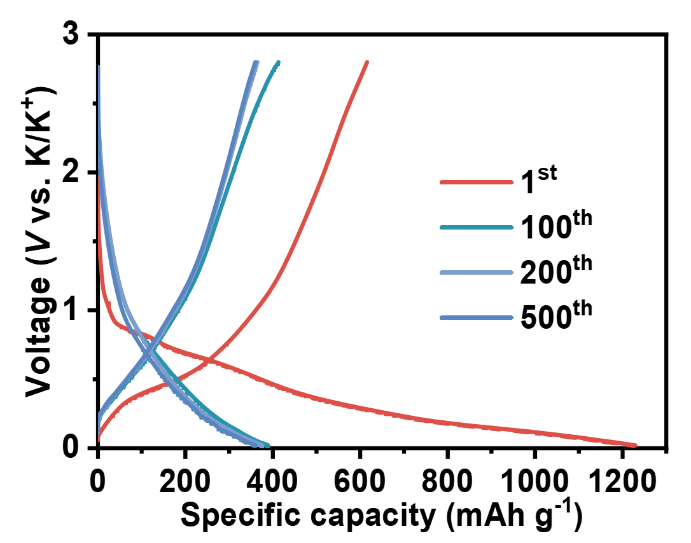


Figure S23: PIBs: Charge/discharge curves of the Fe@FCTF electrode at 0.5 A g^-1^ for different cycles are in Figure 4e.


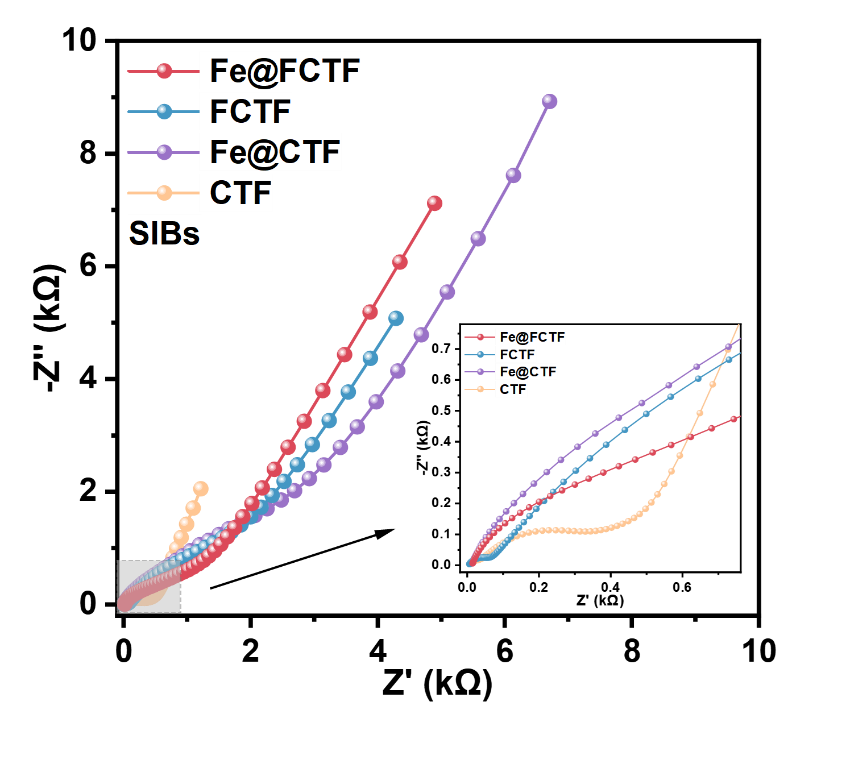


Figure S24: The Nyquist plots for the Fe@FCTF and other CTFs for SIBs.


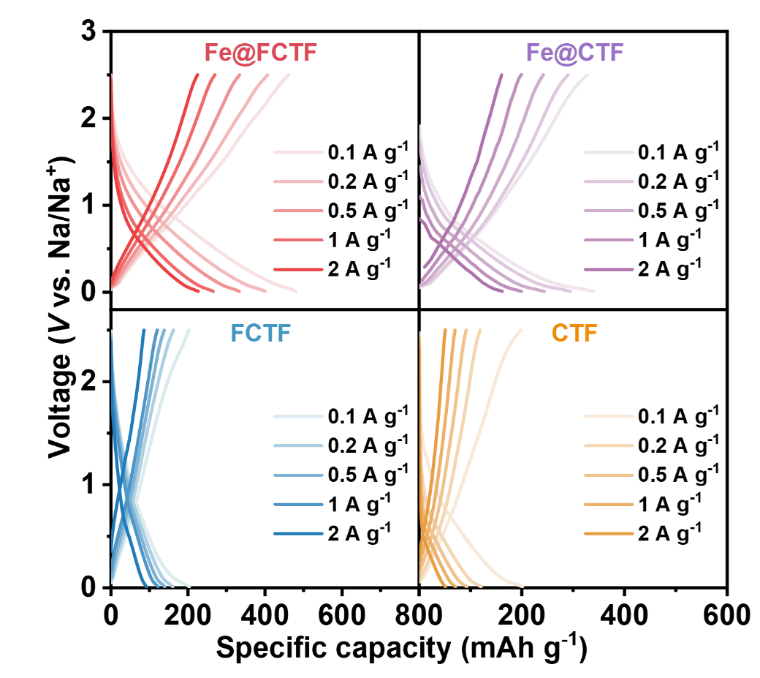


Figure S25: SIBs: Charge/discharge curves under different current densities of the Fe@FCTF and other CTFs.


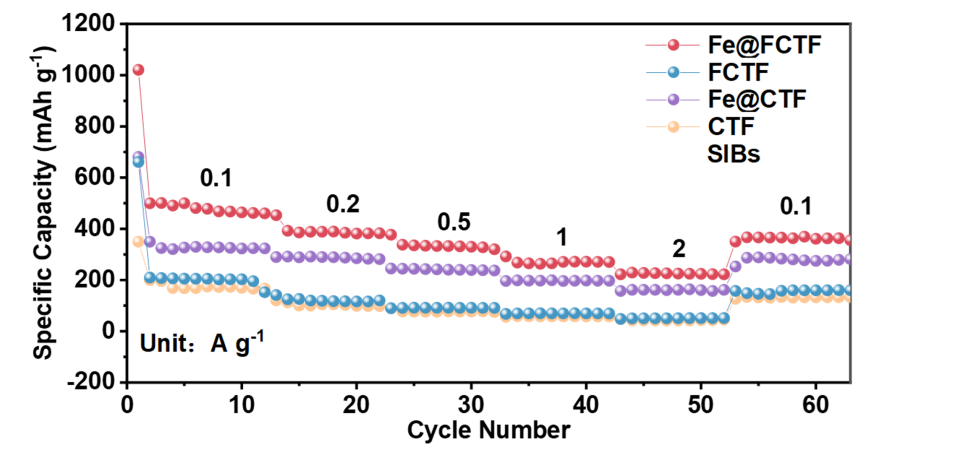


Figure S26: SIBs: The rate capability of Fe@FCTF and other CTFs from 0.1-2 A g^-1^.


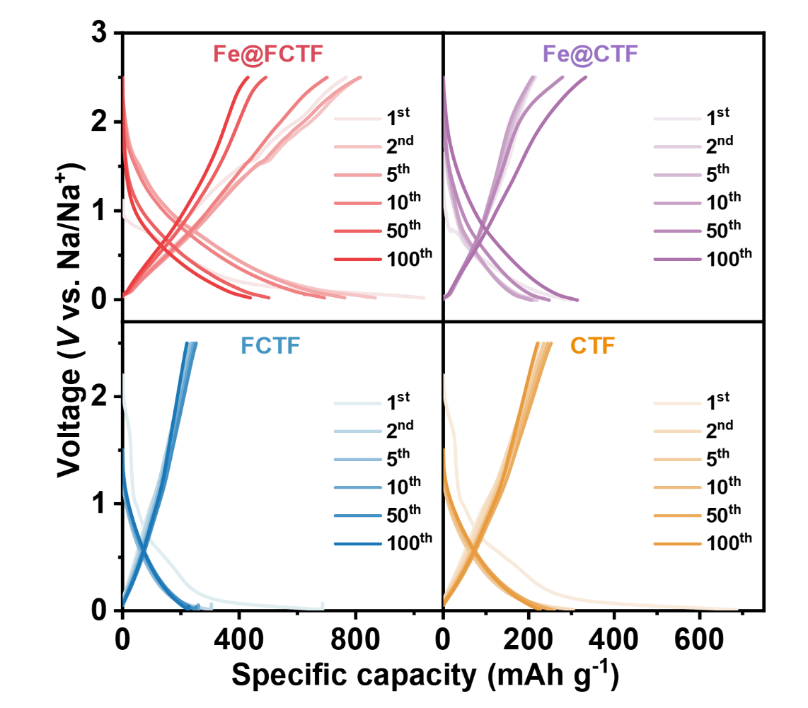


Figure S27: SIBs: Charge/discharge curves of the Fe@FCTF and other CTFs electrodes at 0.1 A g^-1^ for different cycles are in Figure 4i.


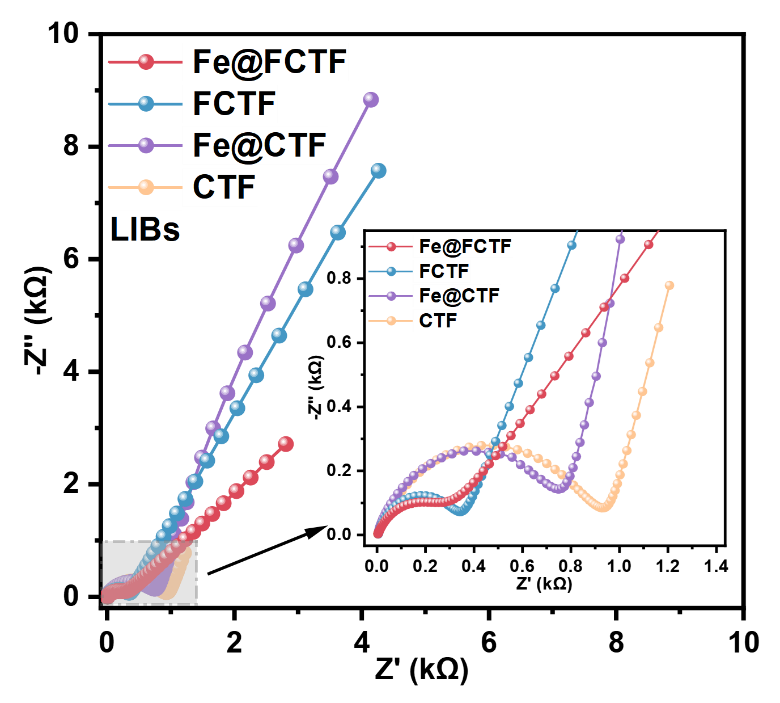


Figure S28: The Nyquist plots for the Fe@FCTF and other CTFs for LIBs.


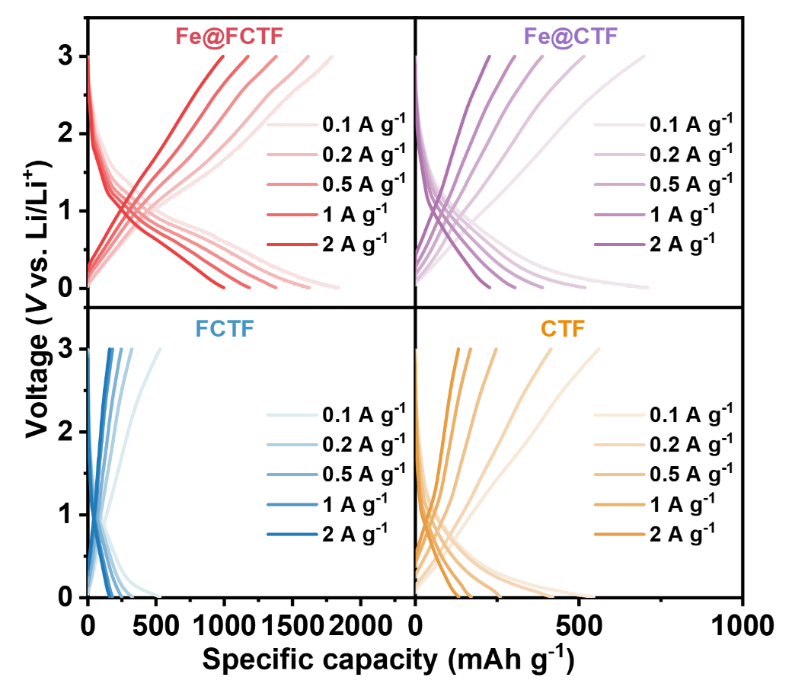


Figure S29: LIBs: Charge/discharge curves under different current densities of the Fe@FCTF and other CTFs.


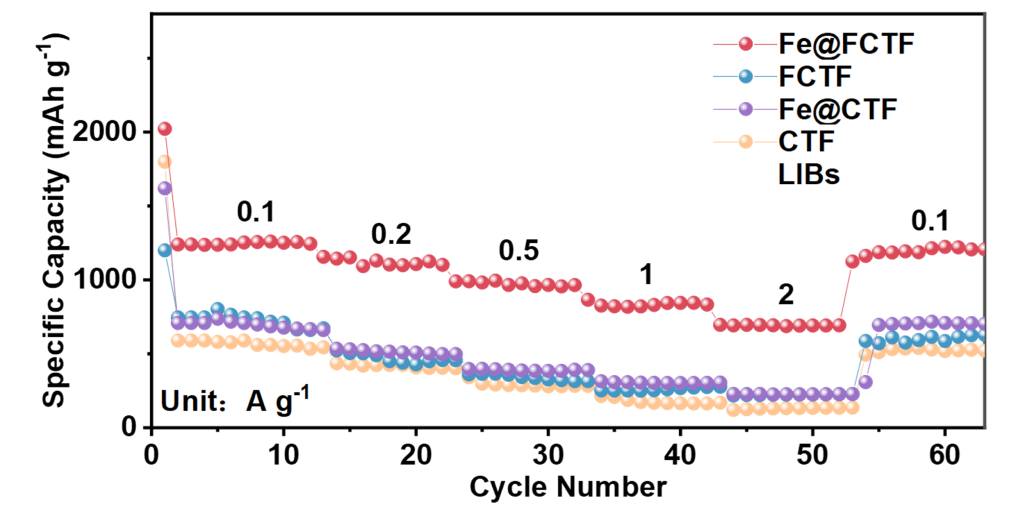


Figure S30: LIBs: The rate capability of Fe@FCTF and other CTFs from 0.1-2 A g^-1^.


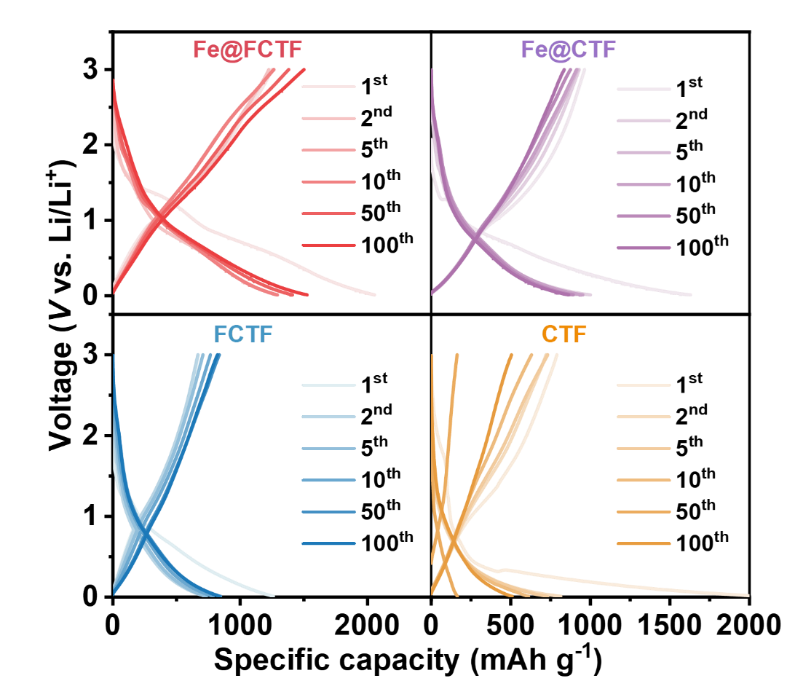


Figure S31: LIBs: Charge/discharge curves of the Fe@FCTF and other CTFs electrodes at 0.1 A g^-1^ for different cycles are in Figure 4m.


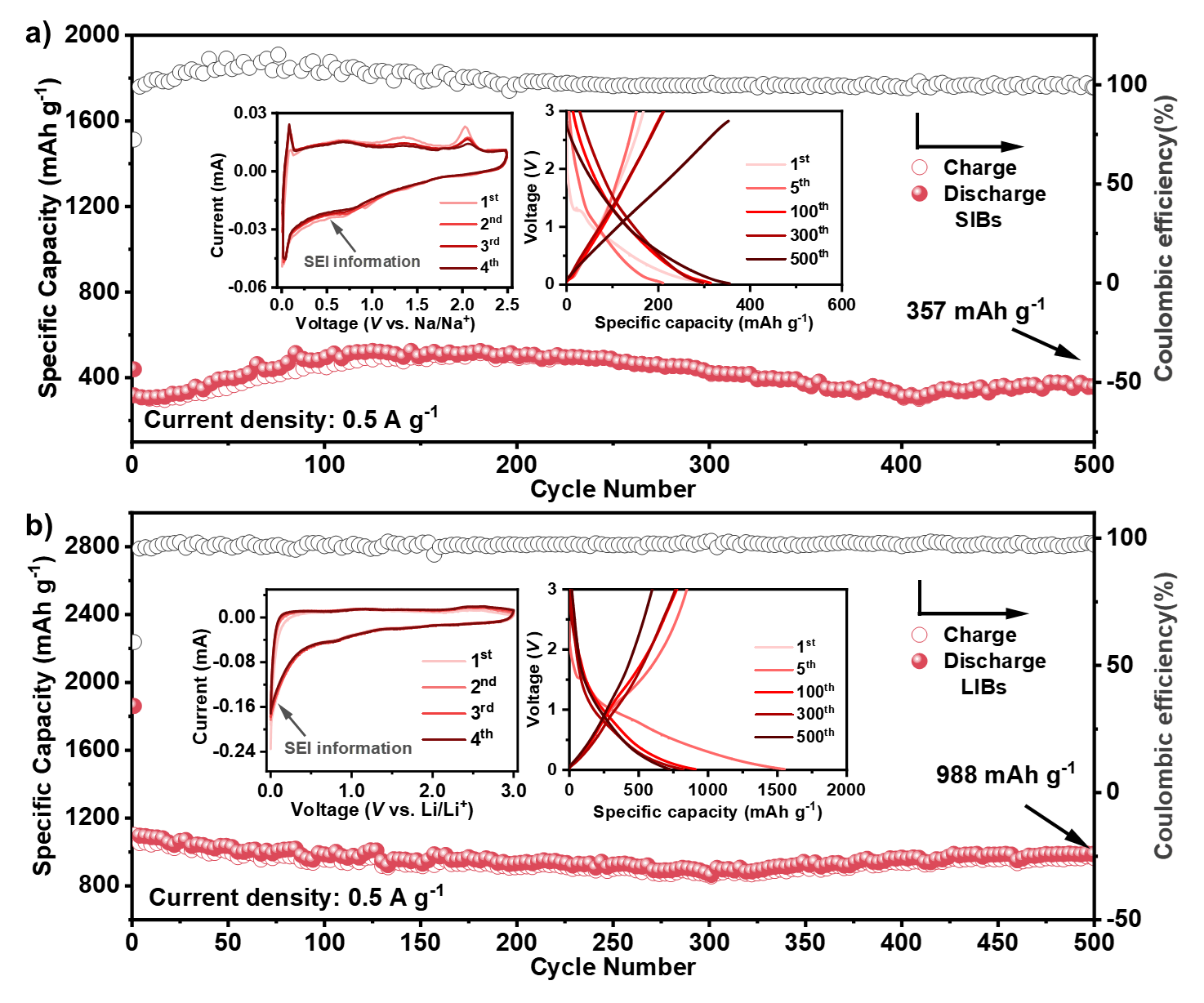


Figure S32: Electrochemical performances of Fe@FCTF for SIBs and LIBs: a) Long cycling stability of the Fe@FCTF electrode at 0.5 A g^-1^ for SIBs. Insert CV curves of Fe@FCTF at the first four cycles at 0.1 mV s^-1^ for SIBs (left) and charge/discharge curves of the Fe@FCTF electrode at 0.5A g^-1^ for different cycles for SIBs (right). b) Long cycling stability of the Fe@FCTF electrode at 0.5A g^-1^ for LIBs. Insert CV curves of Fe@FCTF at the first four cycles at 0.1 mV s^-1^ for LIBs (left) and charge/discharge curves of the Fe@FCTF electrode at 0.5A g^-1^ for different cycles for LIBs (right).


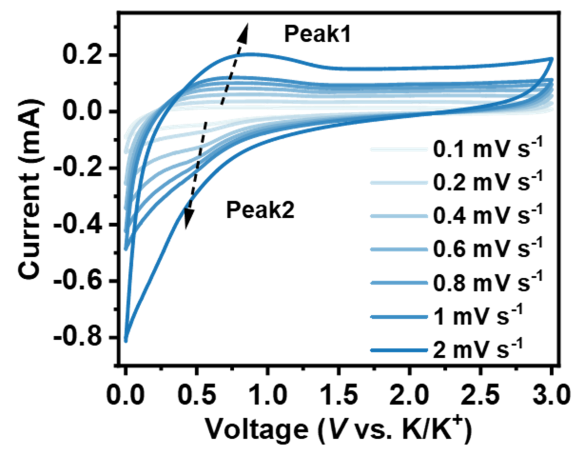


Figure S33: CV plots of FCTF with different scan rates of 0.1 to 2 mV s^-1^ for PIBs.


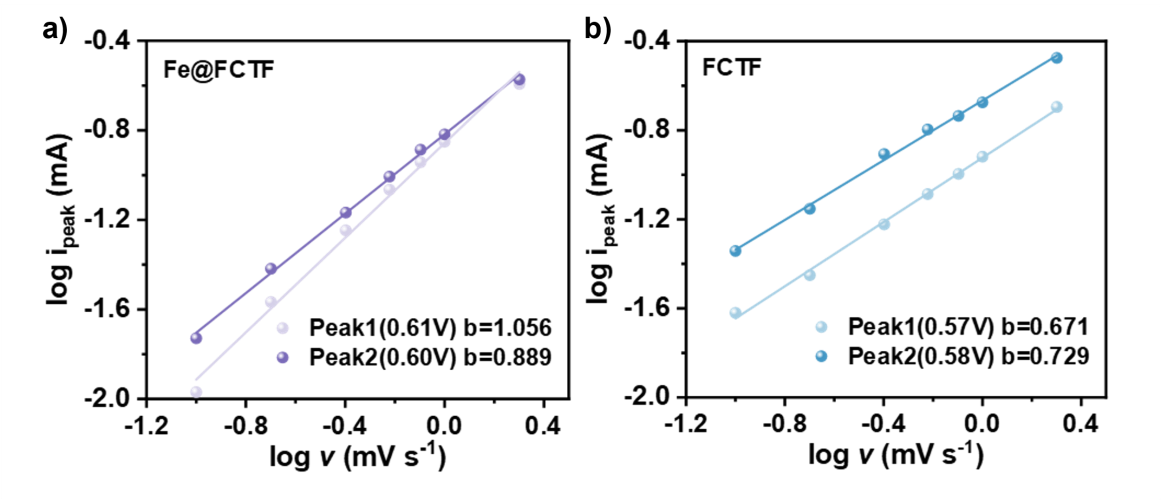


Figure S34: The b-values of Fe@CTF and FCTF at different redox voltages for PIBs.


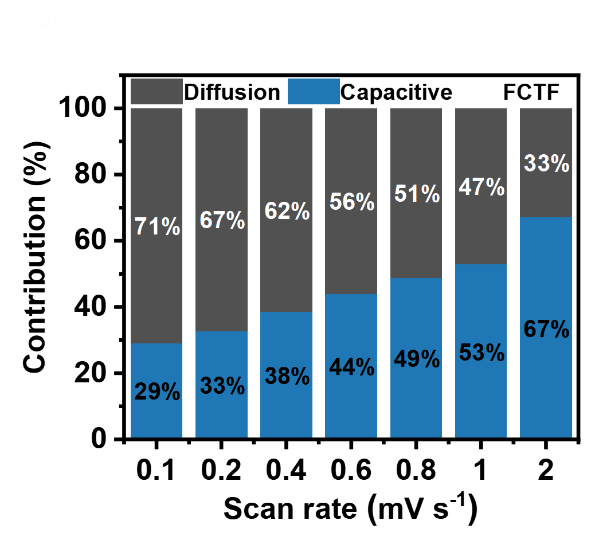


Figure S35: Contribution ratio of capacitive and diffusion-controlled behaviors of FCTF at various scan rates for PIBs.


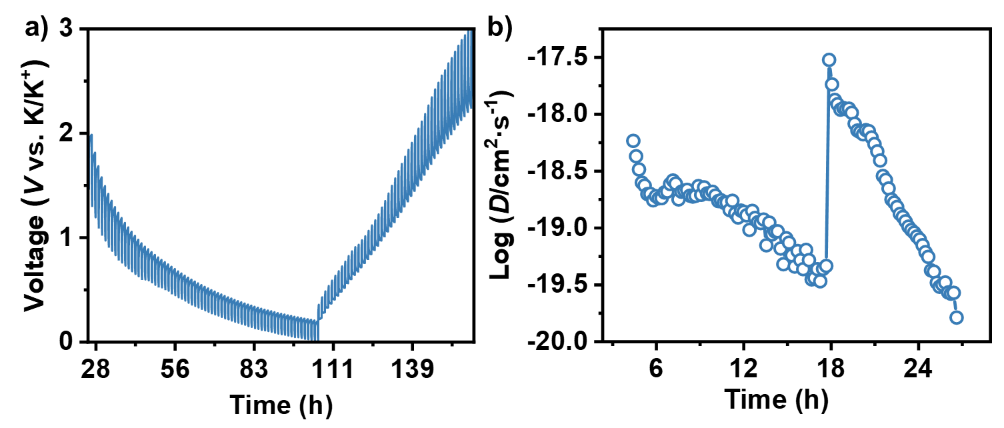


Figure S36: a) GITT curve of FCTF electrode in PIBs. b) The calculated K-ion chemical diffusion coefficients for FCTF.


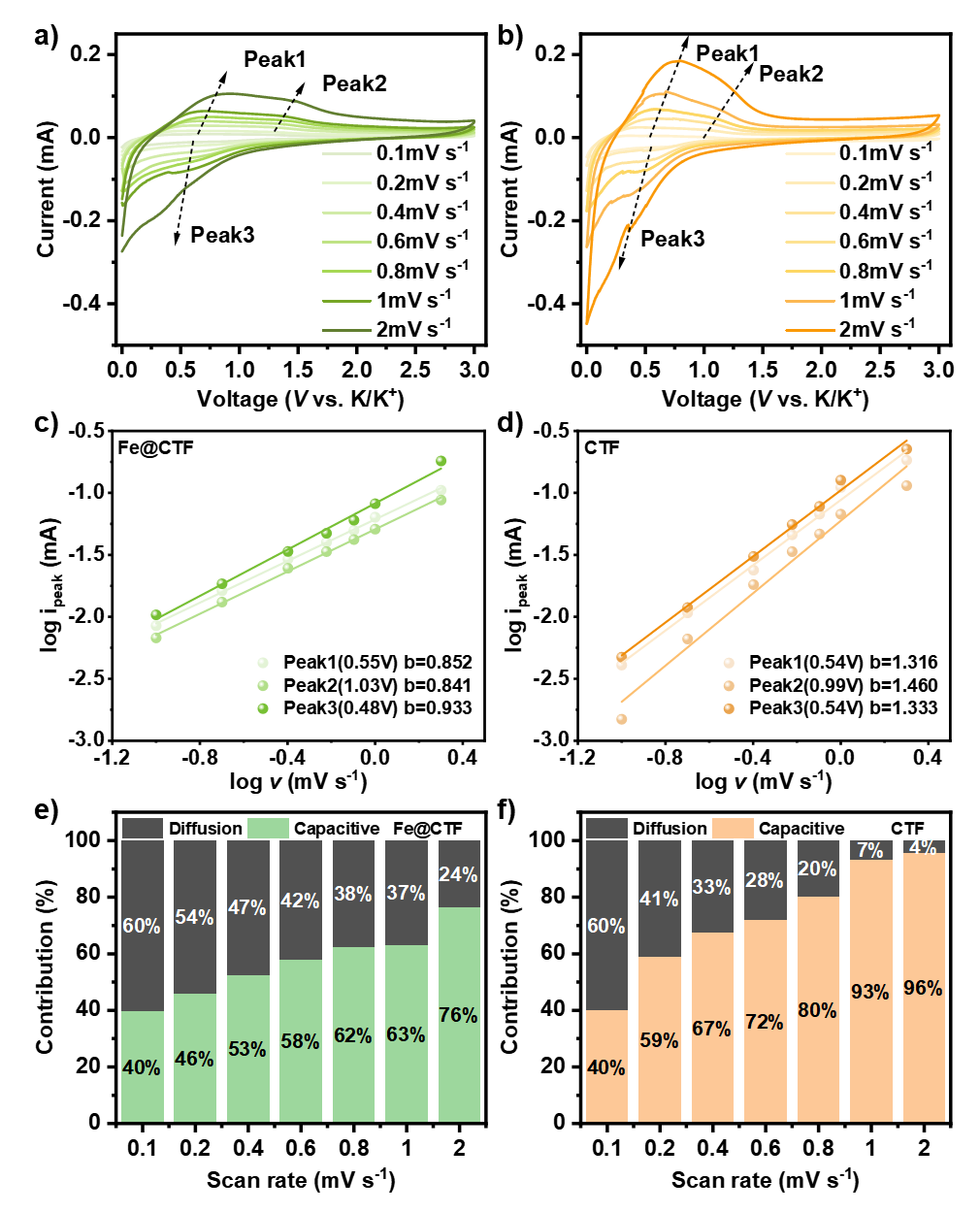


Figure S37: Electrochemical behaviors of the Fe@CTF and CTF for PIBs. a) CV plots of Fe@CTF with different scan rates of 0.1 to 2 mV s^-1^. b) CV plots of CTF with different scan rates of 0.1 to 2 mV s^-1^. c) The b-values of Fe@CTF at different redox voltages. d) The b-values of CTF at different redox voltages. e) Contribution ratio of capacitive and diffusion-controlled behaviors of Fe@CTF at various scan rates. f) Contribution ratio of capacitive and diffusion-controlled behaviors of CTF at various scan rates.


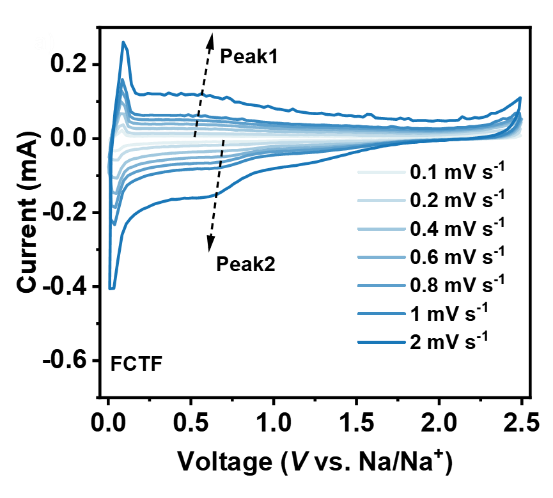


Figure S38: CV plots of FCTF with different scan rates of 0.1 to 2 mV s^-1^ for SIBs.


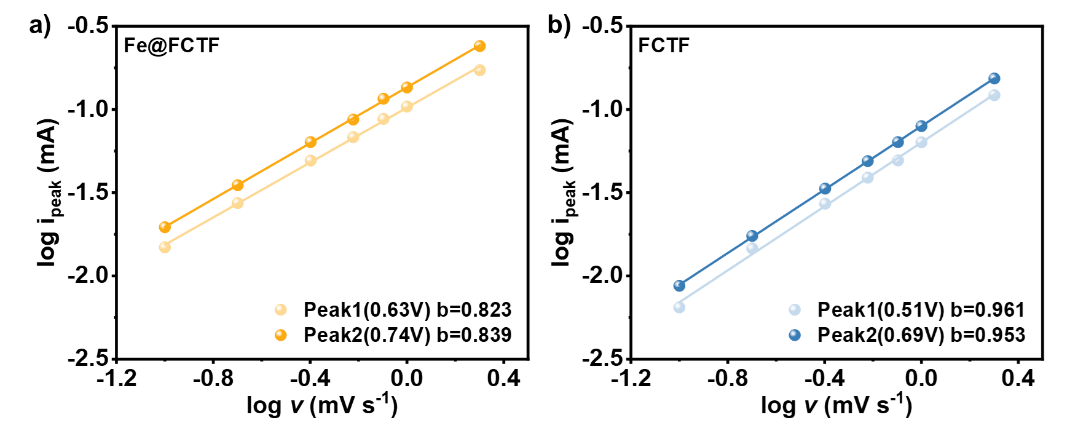


Figure S39: The b-values of Fe@CTF and FCTF at different redox voltages for SIBs.


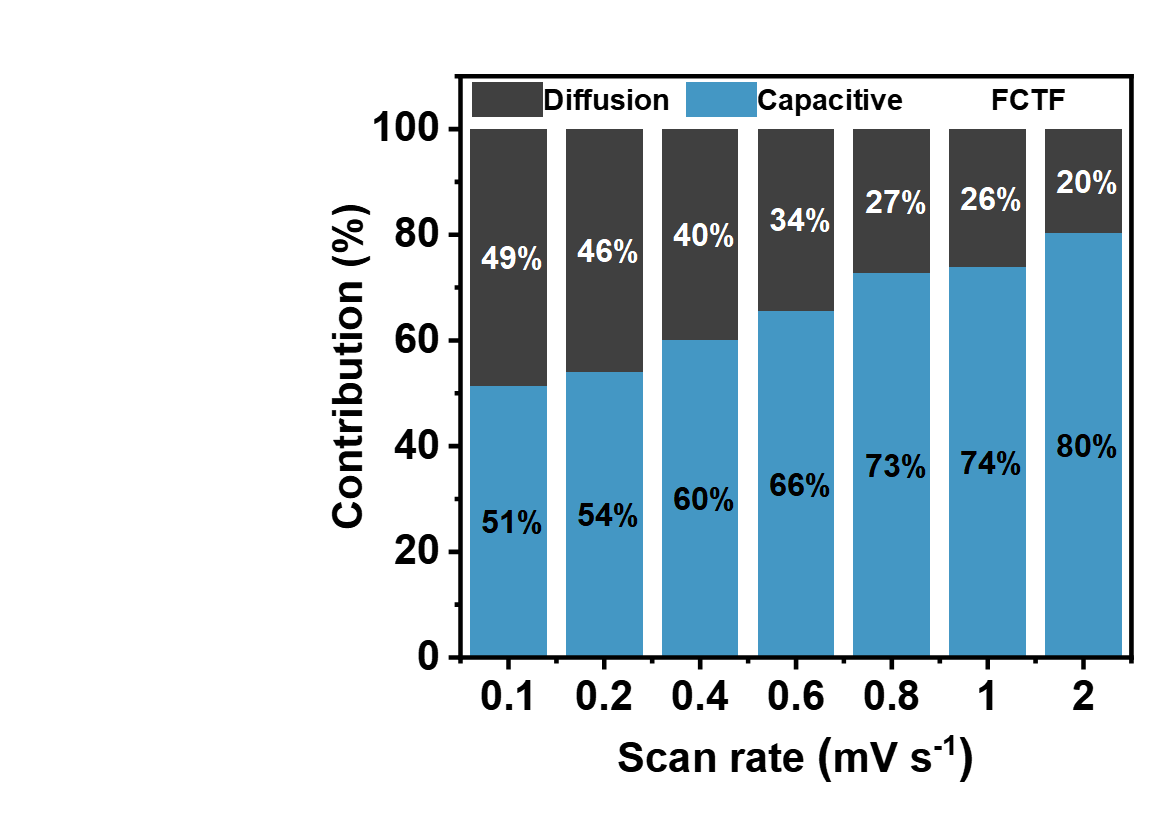


Figure S40: Contribution ratio of capacitive and diffusion-controlled behaviors of FCTF at various scan rates for SIBs.


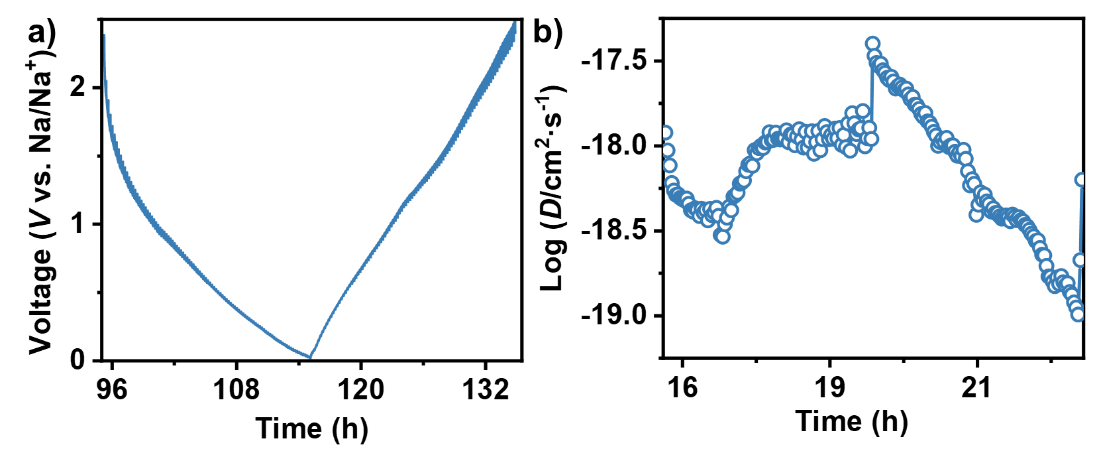


Figure S41: a) GITT curve of FCTF electrode in SIBs. b) The calculated Na-ion chemical diffusion coefficients for FCTF.


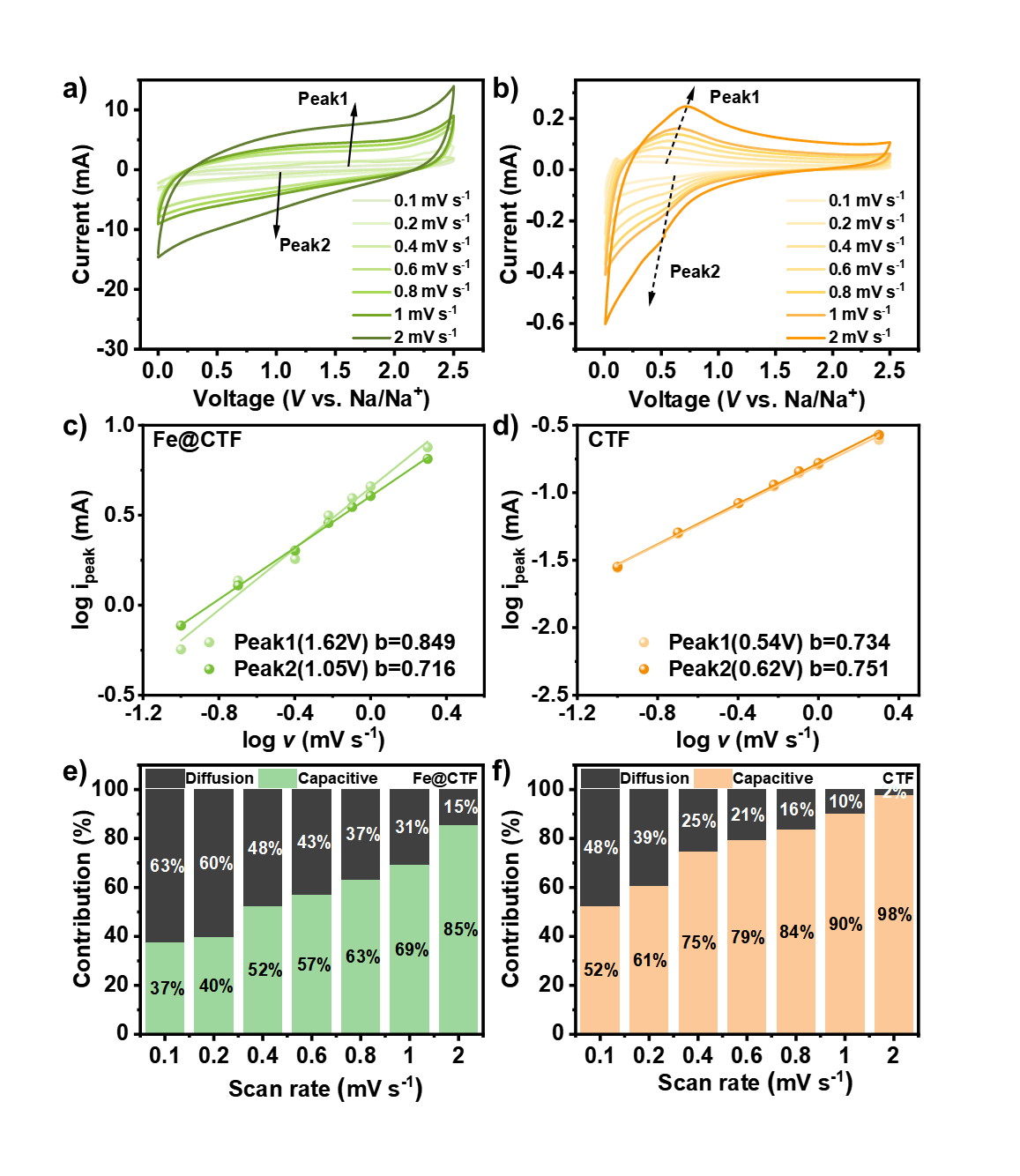


Figure S42: Electrochemical behaviors of the Fe@CTF and CTF for SIBs. a) CV plots of Fe@CTF with different scan rates of 0.1 to 2 mV s^-1^. b) CV plots of CTF with different scan rates of 0.1 to 2 mV s^-1^. c) The b-values of Fe@CTF at different redox voltages. d) The b-values of CTF at different redox voltages. e) Contribution ratio of capacitive and diffusion-controlled behaviors of Fe@CTF at various scan rates. f) Contribution ratio of capacitive and diffusion-controlled behaviors of CTF at various scan rates.


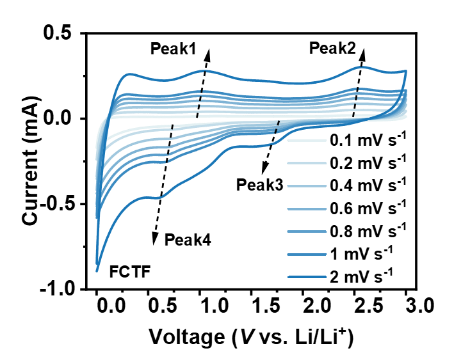


Figure S43: CV plots of FCTF with different scan rates of 0.1 to 2 mV s^-1^ for LIBs.


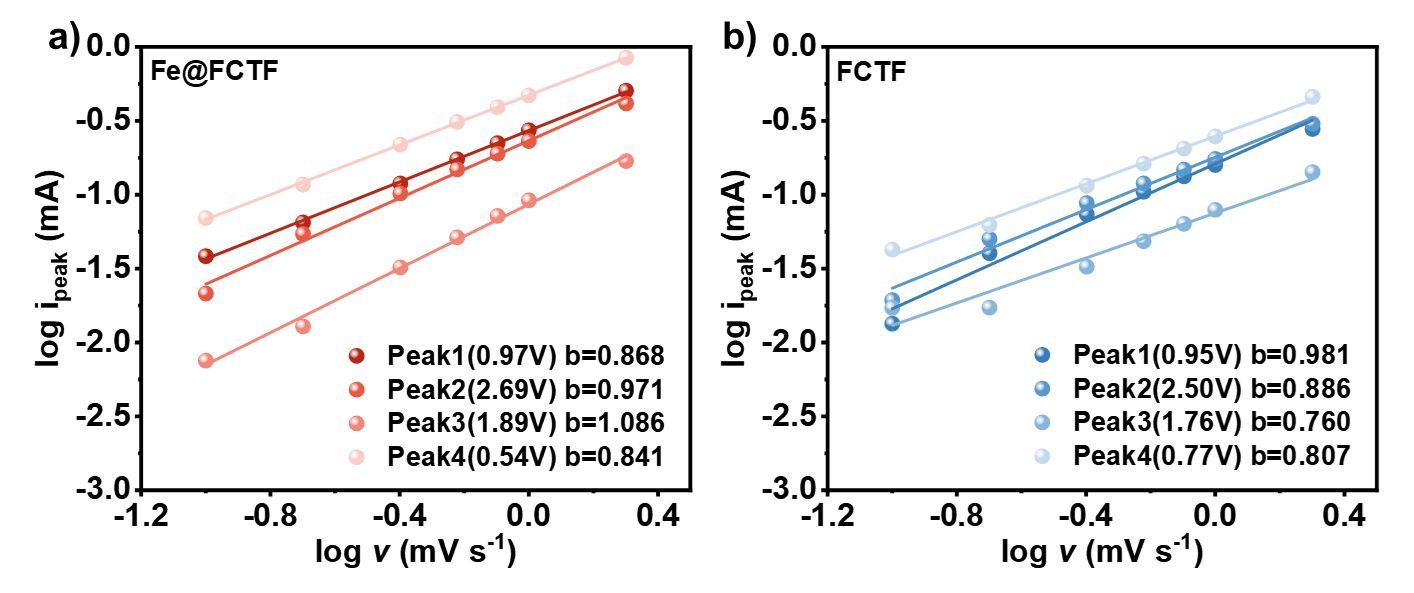


Figure S44: LIBs: The b-values of Fe@CTF and FCTF at different redox voltages.


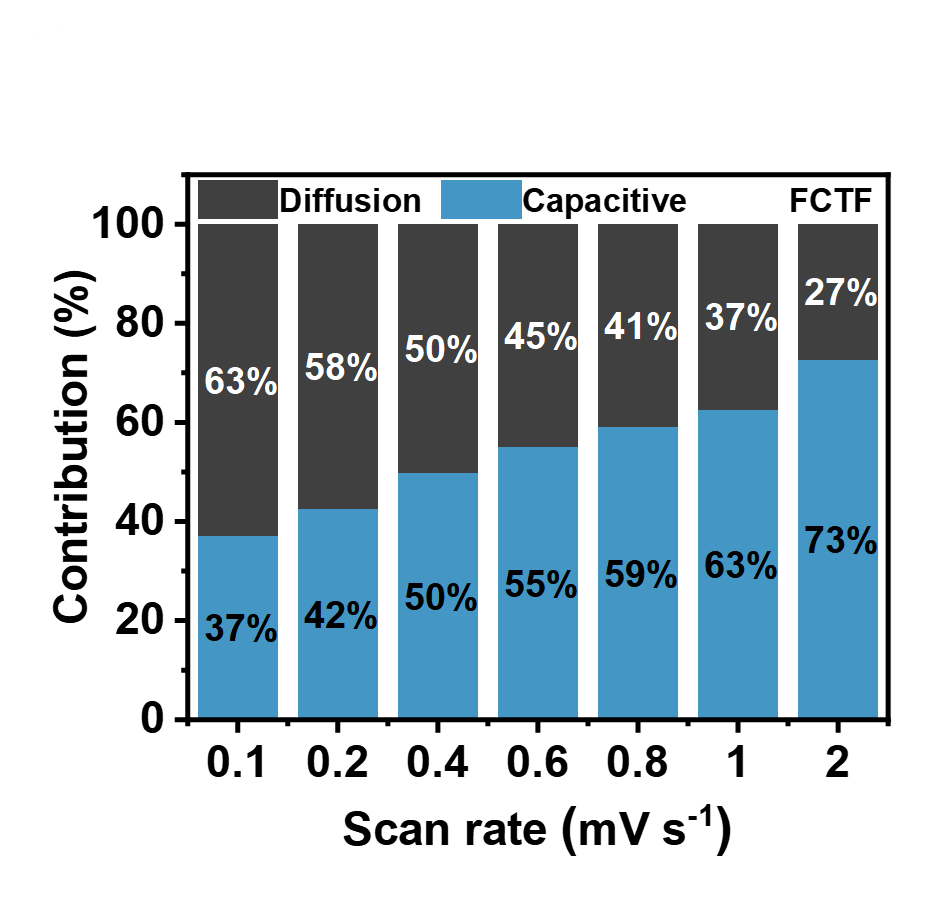


Figure S45: Contribution ratio of capacitive and diffusion-controlled behaviors of FCTF at various scan rates for LIBs.


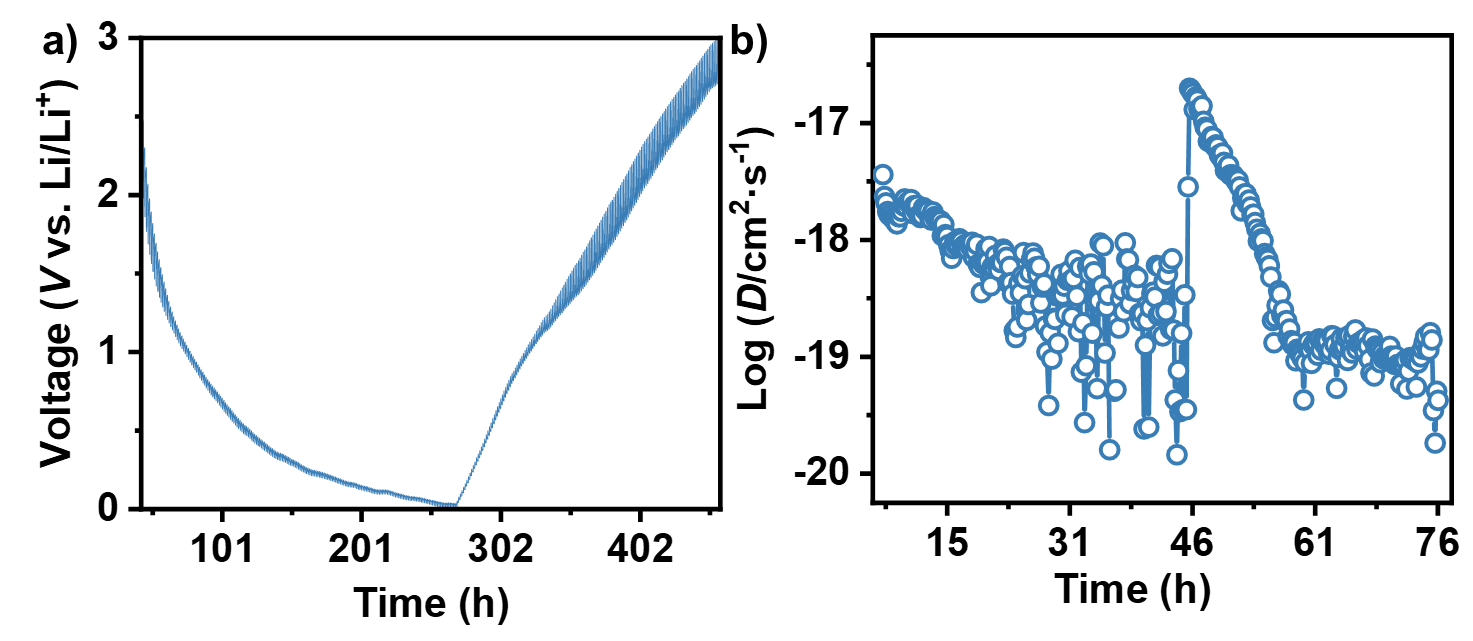


Figure S46: (a) GITT curve of FCTF electrode in LIBs. (b) The calculated Li-ion chemical diffusion coefficients for FCTF.


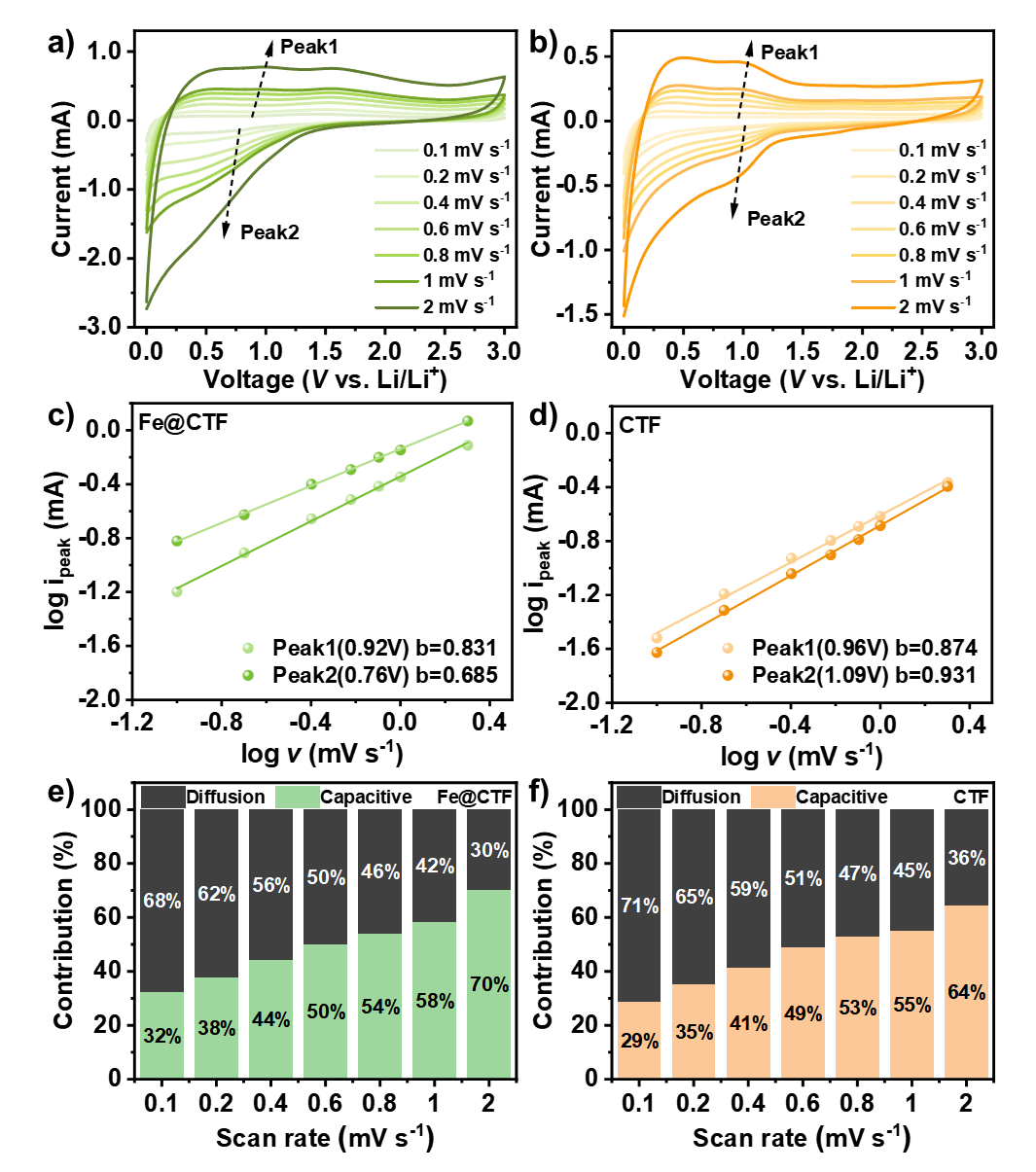


Figure S47: Electrochemical behaviors of the Fe@CTF and CTF for LIBs. a) CV plots of Fe@CTF with different scan rates of 0.1 to 2 mV s^-1^. b) CV plots of CTF with different scan rates of 0.1 to 2 mV s^-1^.c ) The b-values of Fe@CTF at different redox voltages. d) The b-values of CTF at different redox voltages. e) Contribution ratio of capacitive and diffusion-controlled behaviors of Fe@CTF at various scan rates. f) Contribution ratio of capacitive and diffusion-controlled behaviors of CTF at various scan rates.


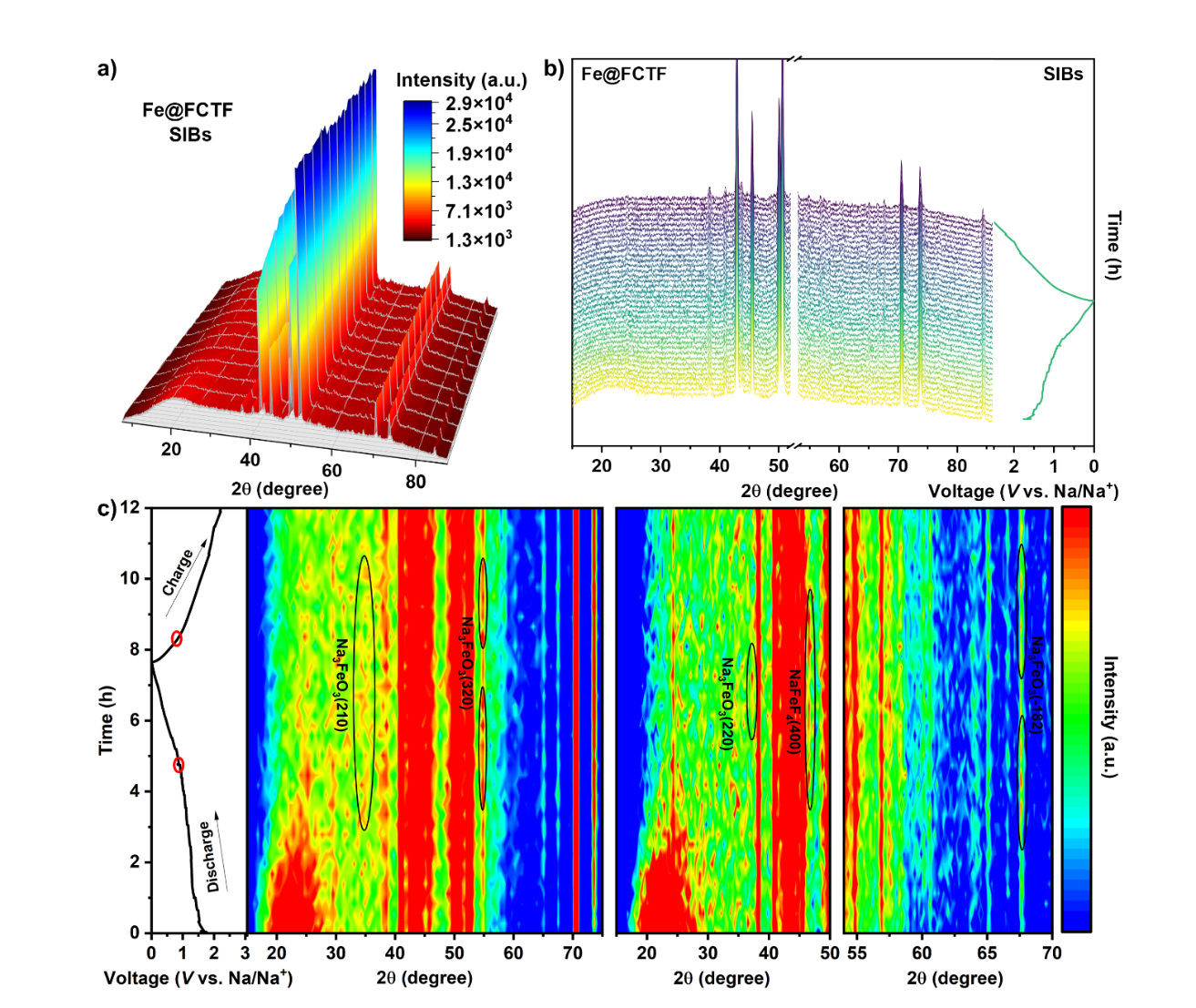


Figure S48: In SIBs: In situ XRD patterns of Fe@FCTF and the associated galvanostatic curve.


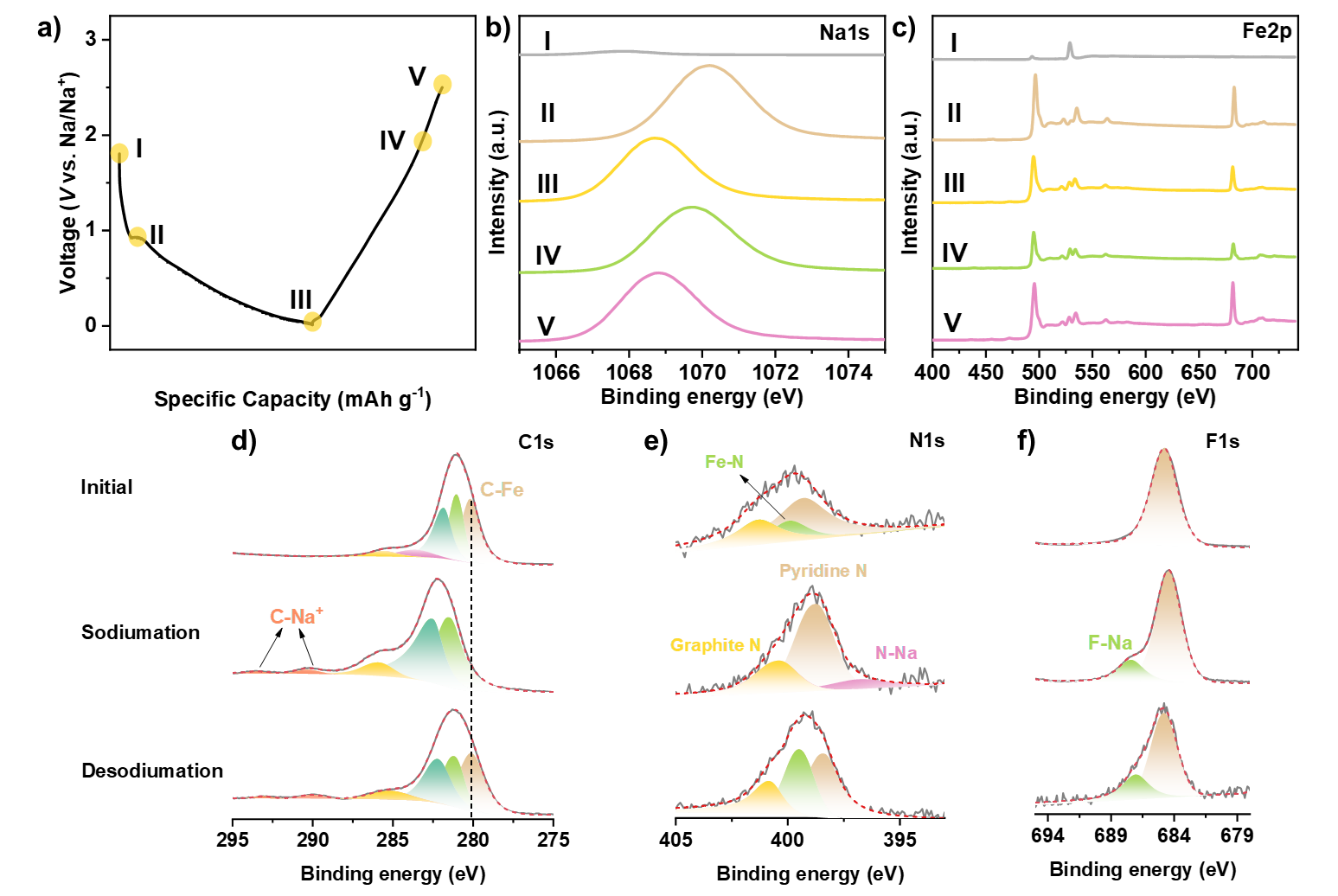


Figure S49: In SIBs: a) Representative charge/discharge curve of Fe@FCTF at a current density of 100 mA g^-1^. I indicates an open circuit state. II, and III indicate that the initial state is discharged to 0.9 V and 0.01 V (vs. Na/Na^+^), while IV and V indicate that the initial state is charged to 1.9 V and 2.5 V (vs. Na/Na^+^). Ex-situ XPS analysis of b) Na1s, c) Fe2p, d) C1s, e) N1s and f) F1s regions.


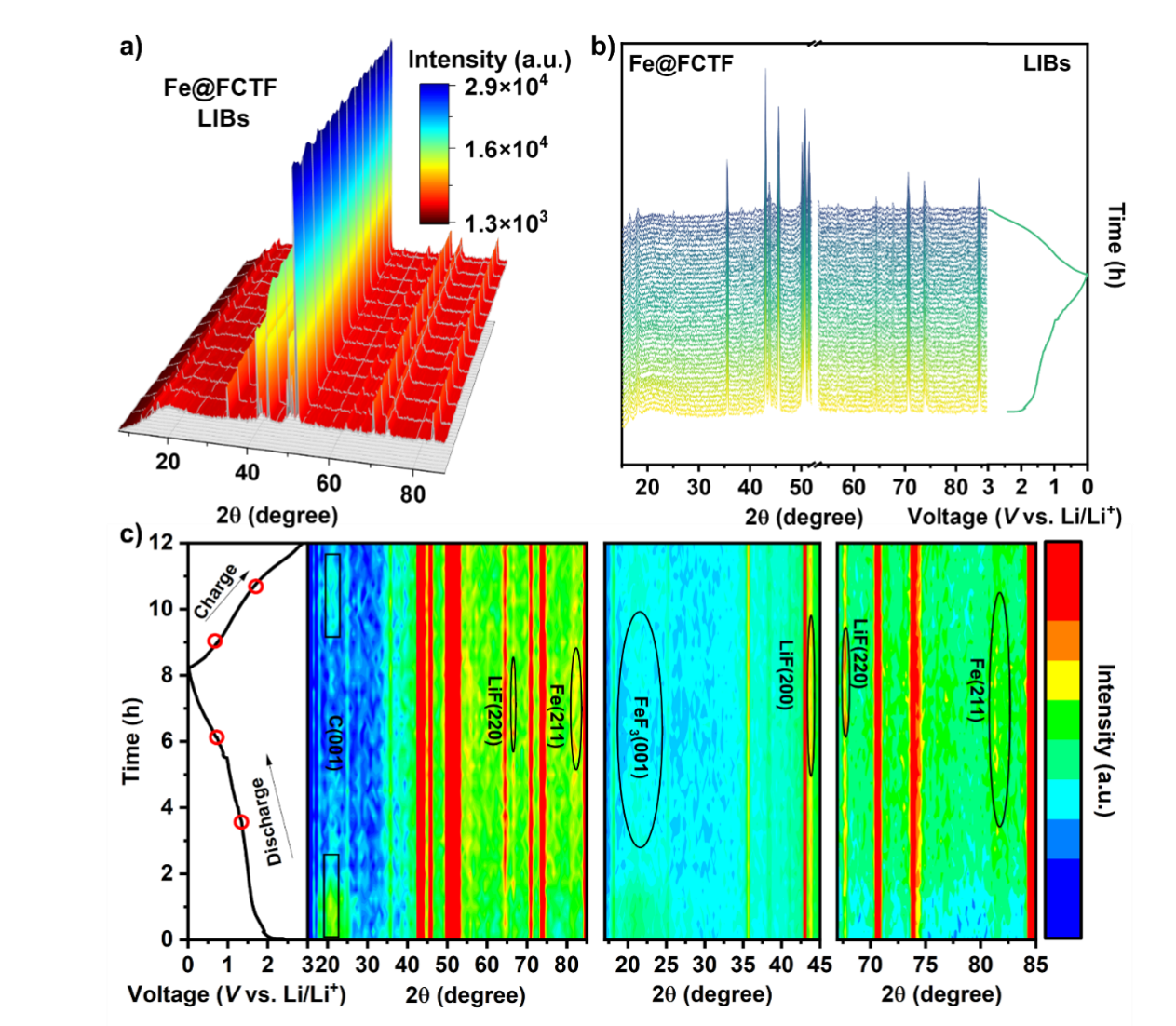


Figure S50: In LIBs: In situ XRD patterns of Fe@FCTF and the associated galvanostatic curve.


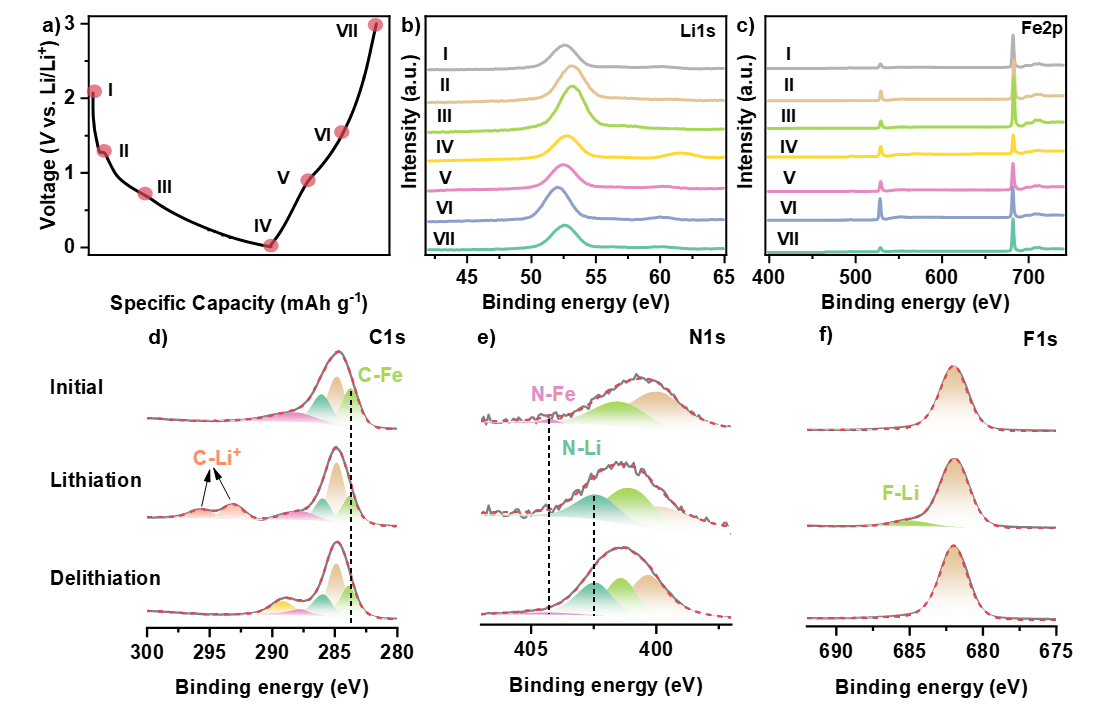


Figure S51: In LIBs: a) Representative charge/discharge curve of Fe@FCTF at a current density of 100 mA g^-1^. I indicates an open circuit state. II, III, and IV indicate that the initial state is discharged to 1.2, 0.6, and 0.01 V (vs. Li/Li^+^), while V, VI, VII, and VIII indicate that the initial state is charged to 0.7, 1.5, and 3.0 V (vs. Li/Li^+^). Ex-situ XPS analysis of b) Li1s, c) Fe2p, d) C1s, e) N1s and f) F1s regions.


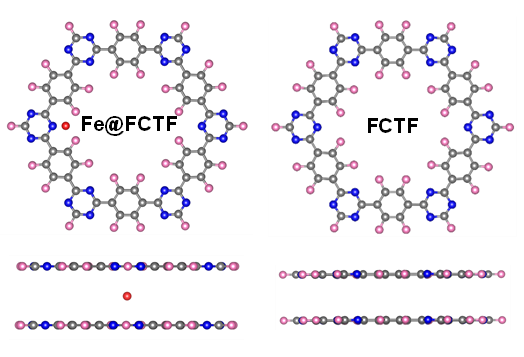


Figure S52: Schematic representation of K^+^ adsorption at Fe@FCTF and FCTF electrodes calculated from simulations (top and side views).


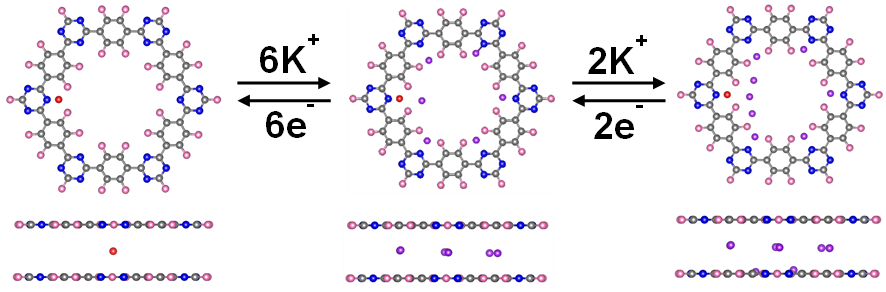


Figure S53: Schematic representation of the optimised Fe@FCTF and FCTF structures.

Table S1: BET test results for CTFs.

| **Sample** | **Fe@FCTF** | **FCTF** | **FeCTF** | **CTF** |
| --- | --- | --- | --- | --- |
| BET Surface Area  (cm^3^ g^-1^) | 450.6 | 383.2 | 109.3 | 336.7 |
| BJH Pore Size  (nm) | 3.0650 | 2.4465 | 5.1700 | 2.0798 |

Table S2: Conductivity test results for Fe@FCTF.

| **Check Numbers** | **X(mm)** | | **Y(mm)** | **Forward Voltage** **(mV)** | | **Reverse Voltage** **(mV)** | **Resistivity**  **(kΩ·cm)** | | **Conductivity**  **(s cm^-1^)** |
| --- | --- | --- | --- | --- | --- | --- | --- | --- | --- |
| **1** | 0 | | 0 | 10.04 | | 10.3 | 4.96 | | 2.02×10^-4^ |
| **2** | 0 | | 0 | 10.78 | | 10.15 | 5.76 | | 1.74×10^-4^ |
| **3** | 0 | | -2.75 | 6.98 | | 6.16 | 3.86 | | 2.59×10^-4^ |
| **4** | 0 | | 0.5 | 6.5 | | 6.76 | 3.92 | | 2.55×10^-4^ |
| **5** | 0 | | -0.5 | 7.67 | | 7.72 | 3.99 | | 2.51×10^-4^ |
| **6** | -2.75 | | 0 | 7.78 | | 7.3 | 3.83 | | 2.61×10^-4^ |
| **7** | 2.75 | | 0 | 9.36 | | 10.05 | 4 | | 2.50×10^-4^ |
| **8** | 0.5 | | 0 | 7.52 | | 7.86 | 3.98 | | 2.51×10^-4^ |
| **9** | -0.5 | | 0 | 8.08 | | 8.32 | 3.99 | | 2.51×10^-4^ |
| **Average**  **Resistivity (kΩ·cm)** | | 4.254 | | | **Average**  **Conductivity (s cm^-1^)** | | | 2.35×10^-4^ | |

Table S3: Conductivity test results for FCTF.

| **Check Numbers** | **X(mm)** | | **Y(mm)** | **Forward Voltage** **(mV)** | | **Reverse Voltage** **(mV)** | **Resistivity**  **(kΩ·cm)** | | **Conductivity**  **(s cm^-1^)** |
| --- | --- | --- | --- | --- | --- | --- | --- | --- | --- |
| **1** | 0 | | 0 | 11.43 | | 2.12 | 6.78 | | 1.47×10^-4^ |
| **2** | 0 | | 0 | 9.17 | | 0.4 | 4.79 | | 2.09×10^-4^ |
| **3** | 0 | | -2.75 | 8.51 | | 1.1 | 4.81 | | 2.08×10^-4^ |
| **4** | 0 | | 0.5 | 8.71 | | 1.02 | 4.87 | | 2.05×10^-4^ |
| **5** | 0 | | -0.5 | 9.74 | | 0.06 | 4.9 | | 2.04×10^-4^ |
| **6** | -2.75 | | 0 | 7.84 | | 1.97 | 4.91 | | 2.04×10^-4^ |
| **7** | 2.75 | | 0 | 10.36 | | 0.81 | 5.59 | | 1.79×10^-4^ |
| **8** | 0.5 | | 0 | 8.86 | | 0.85 | 4.86 | | 2.06×10^-4^ |
| **9** | -0.5 | | 0 | 8.18 | | 1.65 | 4.92 | | 2.03×10^-4^ |
| **Average**  **Resistivity (kΩ·cm)** | | 5.311 | | | **Average**  **Conductivity (s cm^-1^)** | | | 1.94×10^-4^ | |

Table S4: Conductivity test results for Fe@CTF.

| **Check Numbers** | **X(mm)** | | **Y(mm)** | **Forward Voltage** **(mV)** | | **Reverse Voltage** **(mV)** | **Resistivity**  **(kΩ·cm)** | | **Conductivity**  **(s cm^-1^)** |
| --- | --- | --- | --- | --- | --- | --- | --- | --- | --- |
| **1** | 0 | | 0 | 8.93 | | 9.03 | 4.48 | | 2.23×10^-4^ |
| **2** | 0 | | 2.75 | 8.14 | | 8.2 | 4.67 | | 2.14×10^-4^ |
| **3** | 0 | | -2.75 | 6.16 | | 6.39 | 4.78 | | 2.09×10^-4^ |
| **4** | -2.75 | | 0 | 8.56 | | 8.98 | 4.77 | | 2.10×10^-4^ |
| **5** | 2.75 | | 0 | 6.24 | | 6.45 | 4.85 | | 2.06×10^-4^ |
| **6** | 0 | | -0.5 | 6.13 | | 6.48 | 4.81 | | 2.08×10^-4^ |
| **7** | 0 | | 0.5 | 8.07 | | 8.53 | 4.8 | | 2.08×10^-4^ |
| **8** | 0.5 | | 0 | 6.56 | | 6.18 | 4.87 | | 2.05×10^-4^ |
| **9** | -0.5 | | 0 | 9.57 | | 10.04 | 4.81 | | 2.08×10^-4^ |
| **Average**  **Resistivity (kΩ·cm)** | | 4.760 | | | **Average**  **Conductivity (s cm^-1^)** | | | 2.10×10^-4^ | |

Table S5: Conductivity test results for CTF.

| **Check Numbers** | **X(mm)** | | **Y(mm)** | **Forward Voltage** **(mV)** | | **Reverse Voltage** **(mV)** | **Resistivity**  **(kΩ·cm)** | | **Conductivity**  **(s cm^-1^)** |
| --- | --- | --- | --- | --- | --- | --- | --- | --- | --- |
| **1** | 0 | | 0 | 12.3 | | 13.54 | 7.92 | | 1.26×10^-4^ |
| **2** | 0 | | 2.75 | 8.73 | | 9.45 | 4.59 | | 2.18×10^-4^ |
| **3** | 0 | | -2.75 | 6.96 | | 8.56 | 4.76 | | 2.10×10^-4^ |
| **4** | -2.75 | | 0 | 11.27 | | 11.99 | 6.63 | | 1.51×10^-4^ |
| **5** | 2.75 | | 0 | 8.57 | | 9.97 | 4.77 | | 2.10×10^-4^ |
| **6** | 0 | | -0.5 | 8.58 | | 9.81 | 4.7 | | 2.13×10^-4^ |
| **7** | 0 | | 0.5 | 6.71 | | 6.95 | 4.83 | | 2.07×10^-4^ |
| **8** | 0.5 | | 0 | 9.36 | | 9.17 | 4.77 | | 2.10×10^-4^ |
| **9** | -0.5 | | 0 | 8.48 | | 9.18 | 4.83 | | 2.07×10^-4^ |
| **Average**  **Resistivity (kΩ·cm)** | | 5.311 | | | **Average**  **Conductivity (s cm^-1^)** | | | 1.88×10^-4^ | |

Table S6: Atomic percentage of elements on the surface of the sample (Calculated by Thermo Advantage).

| **Element** | **Fe(%)** | **F(%)** | **O(%)** | **N(%)** | **C(%)** | **Cl(%)** |
| --- | --- | --- | --- | --- | --- | --- |
| **Fe@FCTF** | 30.81 | 16.66 | 23.62 | 9.97 | 12.79 | 6.15 |
| **FCTF** | - | 34.97 | 20.67 | 21.69 | 20.94 | 1.73 |
| **Fe@CTF** | 26.39 | - | 32.72 | 10.16 | 22.19 | 8.54 |
| **CTF** | - | - | 31.81 | 32.31 | 32.67 | 3.21 |

Table S7: C1s split-peak area ratio (Calculated by Thermo Avantage).

| **Element** | **Covalent**  **C-F(%)** | **Semi-ionic**  **C-F(%)** | **-COOH**  **(%)** | **-C=N**  **(%)** | **-C=C**  **(%)** | **-C-C**  **(%)** | **C-Fe**  **(%)** |
| --- | --- | --- | --- | --- | --- | --- | --- |
| **Fe@FCTF** | 2.04 | 5.40 | - | 4.06 | 6.55 | 22.77 | 59.18 |
| **FCTF** | 5.10 | 11.16 | - | 15.08 | 29.21 | 39.46 | - |
| **Fe@CTF** | - | - | 3.63 | 14.04 | 17.14 | 51.22 | 13.97 |
| **CTF** | - | - | 3.60 | 34.28 | 26.75 | 35.36 | - |

Table S8: Fe2p split-peak area ratio (Calculated by Thermo Advantage).

| **Element** | **Fe-N**  **(%)** | **Fe^2+^2p_3/2_**  **(%)** | **Fe^3+^2p_3/2_**  **(%)** | **Sat.**  **(%)** | **Fe-N**  **(%)** | **Fe^2+^2p_1/2_**  **(%)** | **Fe^3+^2p_1/2_**  **(%)** | **Sat.**  **(%)** |
| --- | --- | --- | --- | --- | --- | --- | --- | --- |
| **Fe@FCTF** | 27.63 | 12.92 | 7.98 | 18.95 | 8.75 | 16.92 | 3.49 | 3.35 |
| **Fe@CTF** | 10.25 | 38.46 | 11.28 | 4.56 | 8.12 | 19.41 | 5.59 | 2.35 |

Table S9: Electrochemical performance of representative CTF and organic anode materials for PIBs.

| **Electrode** | **Low-rate**  **capacity**  **(mAh g^-1^)** | **Reversible**  **capacity**  **in**  **low-rate**  **(mAh g^-1^)** | **High-rate**  **capacity**  **(mAh g^-1^)** | **Reversible**  **capacity**  **in**  **high-rate**  **(mAh g^-1^)** | **Capacitance contribution rate**  **（%）** | **Ion diffusion coefficient**  **(D/cm^2^·s^-1^)** |
| --- | --- | --- | --- | --- | --- | --- |
| **Fe@FCTF** | 169 | 167 | 655 | 654 | 86 | 10^-17^-10^-18^ |
| **FCTF** | 75 | 75 | 200 | 198 | 67 | 10^-18^-10^-19^ |
| **Fe@CTF** | 74 | 73 | 286 | 251 | 77 | - |
| **CTF** | 32.1 | 32 | 113 | 109 | 96 | - |

Table S10: Electrochemical performance of representative CTF and organic anode materials for SIBs.

| **Electrode** | **Low-rate**  **capacity**  **(mAh g^-1^)** | **Reversible**  **capacity**  **in**  **low-rate**  **(mAh g^-1^)** | **High-rate**  **capacity**  **(mAh g^-1^)** | **Reversible**  **capacity**  **in**  **high-rate**  **(mAh g^-1^)** | **Capacitance contribution rate**  **（%）** | **Ion diffusion coefficient**  **(D/cm^2^·s^-1^)** |
| --- | --- | --- | --- | --- | --- | --- |
| **Fe@FCTF** | 498 | 496 | 223 | 221 | 73 | 10^-16^-10^-17^ |
| **FCTF** | 207 | 201 | 51 | 50 | 80 | 10^-17^-10^-18^ |
| **Fe@CTF** | 335 | 332 | 162 | 163 | 85 | - |
| **CTF** | 174 | 174 | 43 | 42 | 98 | - |

Table S11: Electrochemical performance of representative CTF and organic anode materials for LIBs.

| **Electrode** | **Low-rate**  **capacity**  **(mAh g^-1^)** | **Reversible**  **capacity**  **in**  **low-rate**  **(mAh g^-1^)** | **High-rate**  **capacity**  **(mAh g^-1^)** | **Reversible**  **capacity**  **in**  **high-rate**  **(mAh g^-1^)** | **Capacitance contribution rate**  **（%）** | **Ion diffusion coefficient**  **(D/cm^2^·s^-1^)** |
| --- | --- | --- | --- | --- | --- | --- |
| **Fe@FCTF** | 1833 | 1831 | 992 | 992 | 84 | 10^-17^-10^-18^ |
| **FCTF** | 737 | 735 | 226 | 225 | 73 | 10^-18^-10^-20^ |
| **Fe@CTF** | 708 | 708 | 226 | 226 | 70 | - |
| **CTF** | 580 | 579 | 133 | 132 | 64 | - |
